# Modeling and Dynamical Analysis of Virus-Triggered Innate Immune Signaling Pathways

**DOI:** 10.1371/journal.pone.0048114

**Published:** 2012-10-30

**Authors:** Jinying Tan, Ruangang Pan, Lei Qiao, Xiufen Zou, Zishu Pan

**Affiliations:** 1 School of Mathematics and Statistics, Wuhan University, Wuhan, China; 2 College of Science, Huazhong Agricultural University, Wuhan, China; 3 State Key Laboratory of Virology, College of Life Sciences, Wuhan University, Wuhan, China; Institut National de la Santé et de la Recherche Médicale U 872, France

## Abstract

The investigation of the dynamics and regulation of virus-triggered innate immune signaling pathways at a system level will enable comprehensive analysis of the complex interactions that maintain the delicate balance between resistance to infection and viral disease. In this study, we developed a delayed mathematical model to describe the virus-induced interferon (IFN) signaling process by considering several key players in the innate immune response. Using dynamic analysis and numerical simulation, we evaluated the following predictions regarding the antiviral responses: (1) When the replication ratio of virus is less than 1, the infectious virus will be eliminated by the immune system’s defenses regardless of how the time delays are changed. (2) The IFN positive feedback regulation enhances the stability of the innate immune response and causes the immune system to present the bistability phenomenon. (3) The appropriate duration of viral replication and IFN feedback processes stabilizes the innate immune response. The predictions from the model were confirmed by monitoring the virus titer and IFN expression in infected cells. The results suggest that the balance between viral replication and IFN-induced feedback regulation coordinates the dynamical behavior of virus-triggered signaling and antiviral responses. This work will help clarify the mechanisms of the virus-induced innate immune response at a system level and provide instruction for further biological experiments.

## Introduction

In recent years, there has been an explosion of interest in the innate immune response [1]–[5] because it is now known that most infectious pathogens are eliminated through the innate immune response without necessarily requiring the activation of adaptive immunity [6]–[8]. The application of systems biology tools to the innate immune system will enable comprehensive analysis of the complex interactions that maintain the balance between resistance to infection and disease [9], [10].

**Figure 1:**
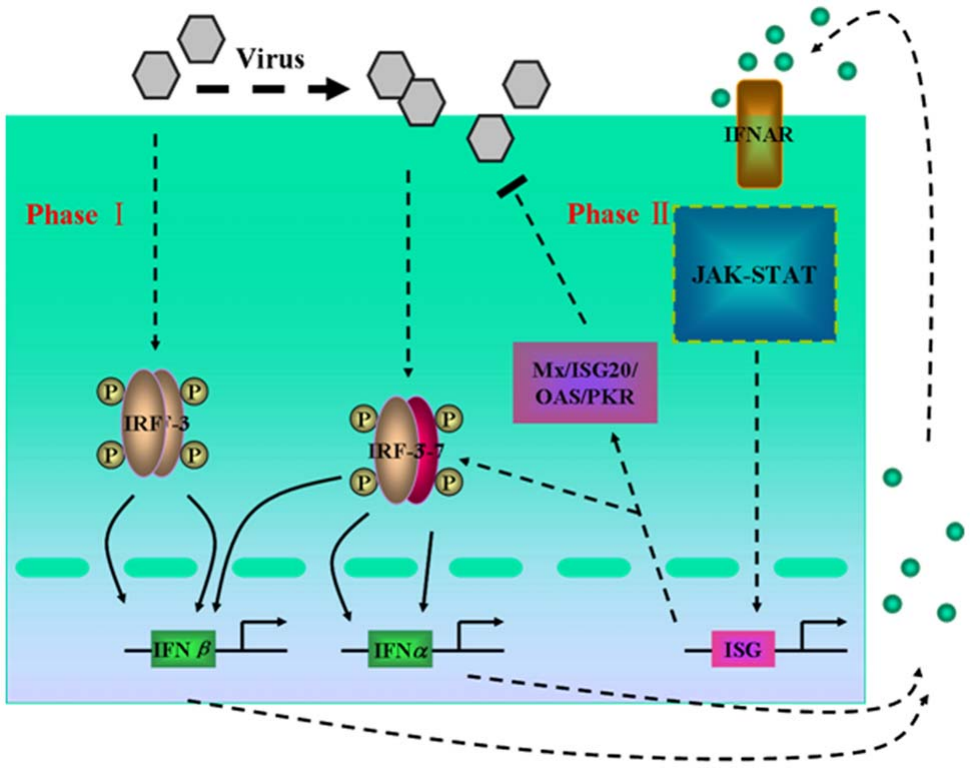
A Schematic reaction diagram of virus-triggered IFN pathways. There are two main phases in type I IFN (IFNα/β) gene expression and regulation by viral infection. In phase I of an infection with viruses, a small amount of IFNβ is induced by the virus. Then, the expressed IFNβ activates the JAK-STAT pathway in phase II, inducing the generation of a large number of IFNα/β and AVPs which inhibits viral replication.

Mathematical modeling and theoretical analyses are increasingly being used to investigate the control mechanism and to identify the coordination of interferon (IFN)-induced JAK-STAT signaling pathways [11]–[15]. The integration of experimental studies with mathematical models of virus-triggered signaling pathways during the primary response was used to explore the effects of viral proteins on type I IFN induction [16], but the late phase of viral infection was not considered. In fact, there are two main phases in type I IFN expression and regulation induced by viral infection [17], [18] (Figure 1). In the early phase of viral infection, IFN-regulatory factor 3 (IRF3) and IRF7 are phosphorylated at specific serine residues, resulting in the homodimerization or heterodimerization of the IRF3 and IRF7. These dimers then translocate to the nucleus and induce the expression of chemokines and small amounts of IFNβ (and IFNα). In the late phase of infection, progeny viruses are produced and released from infected cells. Simultaneously, newly synthesized IFNs bind to the type I IFN receptor (IFNAR) and activate the expression of numerous IFN-stimulated genes (ISGs) via the JAK/STAT pathway. The IFNs then induce the transcription of the IRF7 gene, leading to increases in the expression of the IFNβ and IFNα proteins and, thus, promoting the production of many antiviral proteins (such as Mx, ISG20, OAS and PKR) and immunoactive cytokines. These antiviral components inhibit viral replication and cause apoptosis of infected cells, subsequently resulting in the clearance of the infectious pathogens.

**Figure 2:**
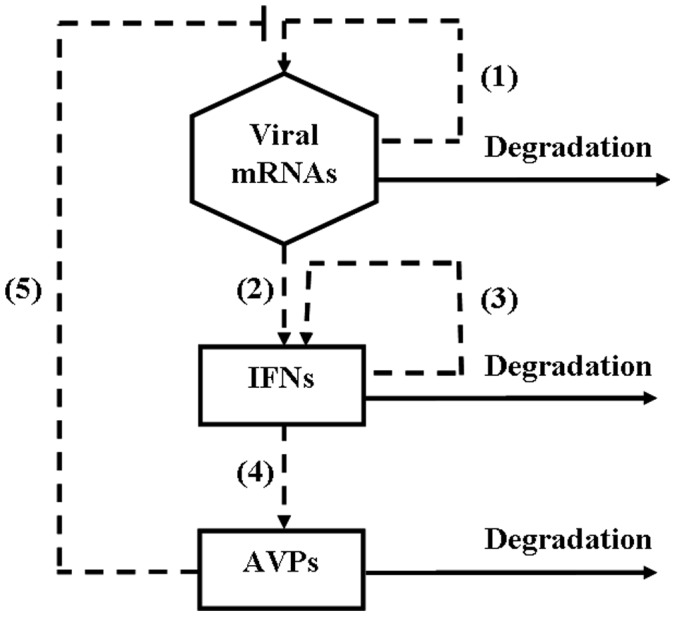
A simplified reaction scheme considered in the mathematical model. There are three main components (viral mRNAs, IFNs and AVPs) and five reactions: (1) the replication of viral mRNAs; (2) the virus-activated IFN expression; (3) the positive feedback of IFNs; (4) the activation of AVPs by IFNs; (5) the inhibition of the virus by AVPs. The dashed lines indicate that there are time delays in the reactions.

To the best of our knowledge, the modeling of the complex process of virus-mediated innate immune response has not been reported in the literature. To better understand the dynamics of the innate immune response and the regulation of the signaling components, we develop a simplified model of virus-activated signaling pathways in the innate immune response. In our model, the interactions between the following components are considered, that is, viral mRNA produced from viral infection (viral mRNAs), type I interferon (IFNs) and antiviral proteins (AVPs) (Figure 2). Dynamical theory and numerical simulations are used to analyze the dynamical features of the virus-triggered innate immune response. Some predictions based on the model are validated by biological experiments.

## Materials and Methods

### Experiments

#### Cell culture, virus infection, and titration

Wild type VISA^+/+^ and knockout VISA^−/−^ MEF cells (Dr. HB Shu, College of Life Sciences, Wuhan University) were used to investigate the dynamics of virus-induced IFN production and the antiviral activity of type I IFNs. The cells were propagated and maintained at 37°C with 5% CO_2_ in Dulbecco’s Modified Eagle Medium (DMEM) (Invitrogen, Carlsbad, CA, USA) supplemented with 10% fetal bovine serum (FBS) (HyClone, Logan, Utah, USA), penicillin (100 units/ml) and streptomycin (100 µg/ml). Vesicular stomatitis virus-expressing fluorescent reporter GFP (VSV*GFP) was used to measure viral replication and IFNβ activity. For viral infection, the cells (5×10^5^ per well) were seeded in 6-well plates and incubated overnight. VSV*GFP was added to each well at 0.05 MOI in 500 µl and incubated for 1 h at 37°C. The wells were washed once with phosphate-buffered saline (PBS) and then 1 ml of growth medium was added. The viral stock was collected at the indicated times postinfection (p.i.). For virus titration, a tissue culture infectious dose 50 (TCID_50_) based on the end-point dilution of the virus at which a cytopathic effect (CPE) is detected in 50% of the cell culture replicates infected by a given amount of virus is employed. The CPE induced by VSV*GFP was observable under a fluorescence microscope at 1–2 days p.i. The VSV*GFP titer was determined using 96-well plates in duplicate from the TCID_50_ of 10-fold serial dilutions (1∶10^1^ to 1∶10^7^) of Opti-MEM (Invitrogen) and was expressed as PFU/ml by calculating the TCID_50_. A fluorescence-activated cell sorting (FACS) assay was further used to quantify VSV*GFP titer in infected cells [19]. In brief, wt VISA^+/+^ and VISA^−/−^ cells were plated in flat-bottom 6-well plates in DMEM at 5×10^5^ cells each well and incubated at 37°C and 5% CO_2_ overnight. 0.05 MOI of VSV*GFP in 500 µl was added to well. After incubation for the indicated times, the cells were washed with cold PBS and incubated with 100 µl of 0.25% Trypsin-EDTA (Gibco, Carlsbad, CA) at 37°C for 5 minutes. The Cells were pelleted by centrifugation at 1600 rpm and washed twice in PBS with 0.1% saponin. Next, the cells were re-suspended in FACS buffer (PBS, 1% FCS and 0.5% sodium azide), and analyzed on the FACSCalibur Flow Cytometer (BD Biosciences, Franklin Lakes, NJ). Virus-positive populations were determined by negatively gating on uninfected samples.

#### VSV-based IFN bioassay

The IFN concentration was measured by a bioassay based on the IFNα/β-mediated VSV growth reduction as previously described [20], [21]. Wt VISA^+/+^ and VISA^−/−^ cells were infected with Sendai virus and at the indicated times, supernatants were collected for measurement of IFNβ concentration. Approximately 1×10^4^ cells were seeded into each well of a 96-well plate and incubated overnight at 5% CO_2_ and 37°C. The cells were then treated either with serial dilutions of standard IFN or serial 10-fold dilutions of IFN-containing samples in 100 µl of growth medium for the indicated times. Subsequently, the growth medium was removed and 1×0.01 MOI of VSV*GFP in 100 µl of infection medium (DMEM with 2% FCS) was added to each well. After 24–48 hrs of further incubation, the cells were observed under a fluorescence microscope and the viral titer was expressed as PFU/ml by counting the TCID_50_.

### Models

#### Mathematical model and nondimensionalization

In this study, the two phases of viral infection were considered. The graphical representation of the mathematical model including the three components and reactions considered is depicted in Figure 2. Upon viral infection, we considered five reactions: (1) the replication of viral mRNAs, (2) the virus-activated IFN expression, (3) the positive feedback of IFNs, (4) the expression of AVPs activated by IFNs and (5) the inhibition of virus replication by AVPs. Because these reactions include the multistep reaction processes which need to take the time to finish, we introduced time delays to describe them for the simplicity. The concentration of reactant 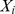 that changes over time can be described by an ordinary differential equation (ODE).
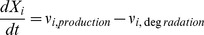
where 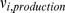 and 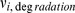 represent the production rate and the degradation rate of reactant 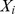, respectively. Based on the law of mass action, we assume that the production rates for three reactions ((1), (2) and (4)) and the degradation rates of three components are linearly proportional to their concentrations. The rates for reactions (3) and (5) are represented using Hill functions. In this paradigm, the dynamics of this network is determined by a system of nonlinear ordinary differential equations (ODEs).
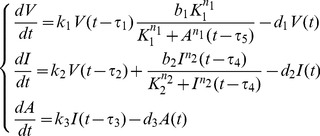
(1)where *V* (mol/L), *I* (mol/L) and *A* (mol/L) are the concentrations of viral mRNAs, IFNs and AVPs, respectively.

In model (1), there are five time delays. Two time delays 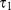 and 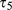 are related to the two processes (1) and (5) that require an intrinsic discrete time to be accomplished. The other three time delays (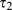, 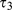 and 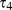) are associated with the three reactions ((2), (3) and (4)) that include the multistep reaction processes not described in detail in the model. The definitions of all parameters in model (1) are described at the first and second columns of Table 1.

**Table 1:** The definitions and values of all parameters in model (1).

Parameters	Process or Description	Range	Reference	Value	Unit
*k*_1_	The kinetic rate constant of the viral replication	–	Estimated	4	h^−1^
*k*_2_	The activation rate constant of IFNs induced by viruses	–	Estimated	0.3	h^−1^
*k*_3_	The activation rate constant of AVPs induced by IFNs	–	Estimated	0.1	h^−1^
*d*_1_	The degradation rate of viral mRNAs	0.1	[22]	0.1	h^−1^
*d*_2_	The degradation rate of IFNs	0.1∼0.7	[23]	0.7	h^−1^
*d*_3_	The degradation rate of AVPs	0.03∼0.35	[24], [25]	0.12	h^−1^
*b*_1_	The maximal production rate of the Hill function of AVPs on the viruses	–	Estimated	10	–
*b*_2_	The maximal production rate of the Hill function of IFN positive feedback	–	Estimated	80	mol/(Lh)
*K*_1_	The inhibition coefficient of the Hill function of AVPs on the viruses	–	Estimated	33	mol/L
*K*_2_	The activation coefficient of the Hill function of IFN positive feedback	–	Estimated	0.1	mol/L
*τ*_1_	The duration of the viral replication	8∼12	[22]	12	h
*τ*_2_	The time required for virus-inducible IFN production and secretion	4∼8	[26]	8	h
*τ*_3_	the time required for IFN-induced AVP production	2∼6	[27]–[29]	7	h
*τ*_4_	the duration of IFN positive feedback	–	Estimated	9	h
*τ*_5_	the delay in AVP-mediated inhibition of virus production	–	Estimated	5	h
*n*_1_	Hill coefficient	–	Estimated	1	–
*n*_2_	Hill coefficient	–	Estimated	1	–

Note: The degradation rate of virus, IFN or AVP amounts to ln2/T_half-life_ [30] and its unit is h^−1^. The half-life of virus is about 7 hour [22], the half-life of IFN is about 1∼7 hour [23], which the half-life of IFNβ is about 1∼3 hour and the half-life of IFNα is about 4∼7 hour, and the half-life of AVP is 2∼24 hour [24], [25], which is in fact the range of the half-life of Mx protein which is a kind of important anti-virus protein.

To make the theoretical analysis convenient, we nondimensionalize the model (1). Time is scaled relative to the rate of degradation of the AVPs (*d*_3_). We make the following substitutions and assume that all of the model parameters are greater than 0 for the actual biological significance.



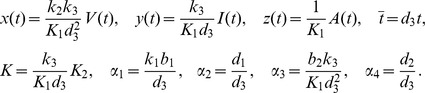



Using 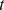 instead of 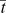 for notational convenience, we obtain the non-dimensional system of equations:



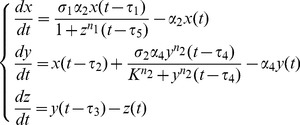
(2)where 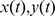 and 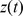 represent the dimensionless concentrations of viral mRNAs, IFNs and AVPs, respectively. Model (2) is determined by five dimensionless parameters *α*_i_ (i = 1,2,3,4) and *K*. *α*_2_ and *α*_4_ correspond to the degradation rates of the virus and IFNs divided by the rate of the degradation of AVPs (time scale), respectively, which are called their relative degradation ratios. *α*_1_ corresponds to the replication rate of the virus divided by the rate of the degradation of the AVPs; *α*_3_ corresponds to the activation rates of IFN positive feedback and the activation ratio of AVPs divided by the rate of the degradation of AVPs. *K* is relative to the production ratio of AVPs.

Let 
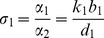
, 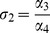 and 
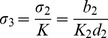
. Then we can view *σ*_1_, *σ_2_* and *σ*_3_ as the replication ratio of the virus, the production ratio of IFN and the relative strength of IFN positive feedback, respectively.

#### Numerical simulation

All numerical simulations are implemented using MATLAB 2009b (The MathWorks, Natick, MA). The system of ordinary differential equations with no delay and delay-differential equations were numerically solved by the subroutine ode45 and dde23 respectively.

#### Steady-state analysis and bifurcation analysis of the model

From a biological point of view, if a system possesses a stable steady state, then this corresponds to a normal biological process. In contrast, the existence of unstable steady states or oscillations corresponds to pathology, *i.e*., an abnormal biological process. To control the viral infection, we seek conditions on the parameters of the signaling process that can guarantee the existence of the steady states.

The steady state concentrations are obtained by solving the system of algebraic equations (2).

When *n*_1_ = *n*_2_ = 1 in system (2), there are three nonnegative steady states: a trivial steady state (O_1_), which represents the absence of all components; a virus-clearance steady state (O_2_), which corresponds to the host eliminating the virus and returning to its normal immune state; and a virus-latent steady state (O_3_), which implies that the virus is coexisting with the host.
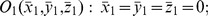


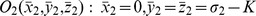


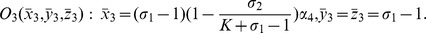
When *n*_1_ = *n*_2_ = 2 in system (2), there are four nonnegative steady states: a trivial steady state (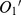), which represents the absence of all components; two virus-clearance steady states (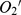
and 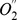), which correspond to the host eliminating the virus and returning to the normal immune state; and a virus-latent steady state (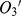), which implies that the virus is coexisting with the host.
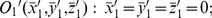


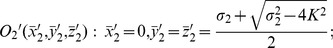


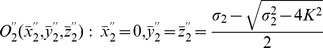



and



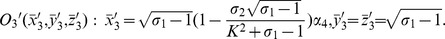



We primarily use the basic stability principle in a dynamic system [31] and the Routh-Hurwitz criterion [32] (see Supporting Information) to analyze the stability of the steady state conditions. We use the Hopf bifurcation theorem [33] to analyze the Hopf bifurcation of the system (2).

## Results

### Comparison between the Simulation and Experimental Viral Replication Results

**Figure 3:**
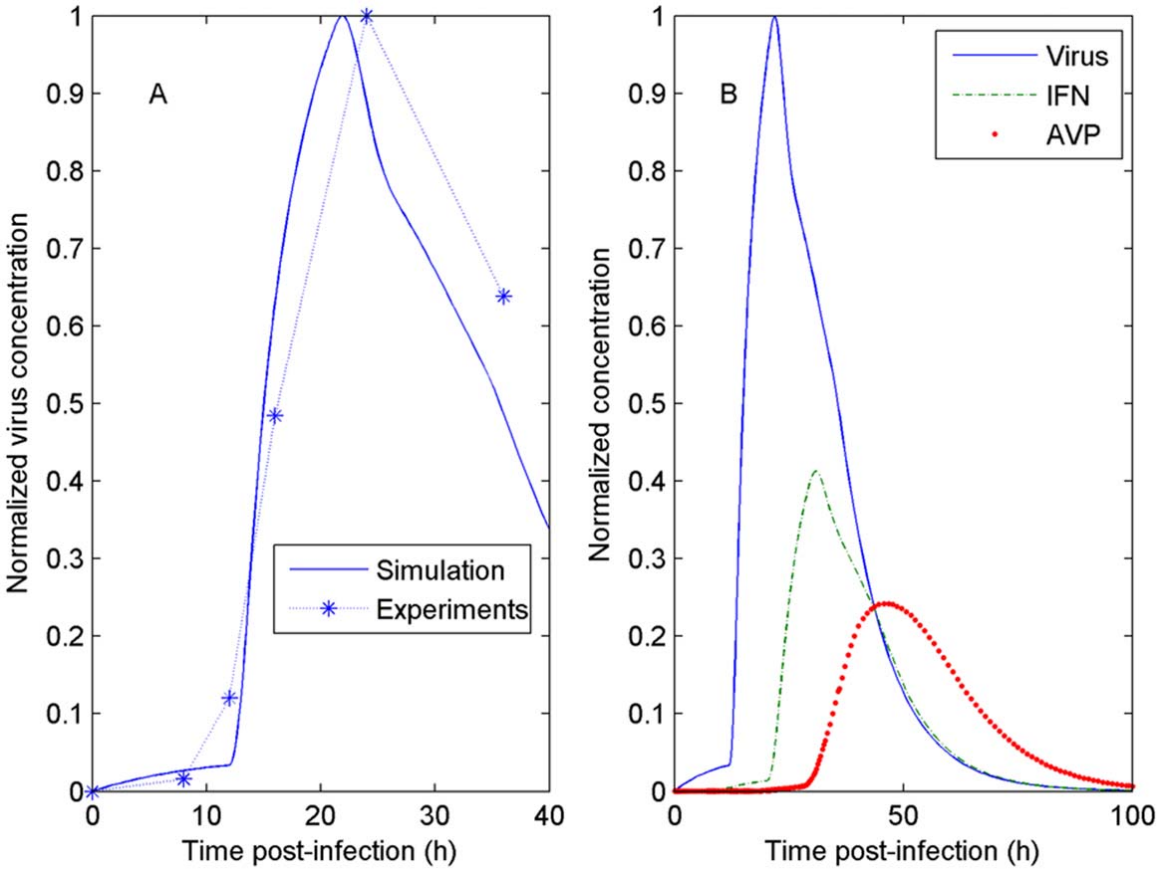
Comparison between the numerical simulation and the experimental results. (A). The time courses of the normalized virus titers from experiments and simulation; (B). The numerical simulation of three components. The initial value is [1000, 10, 2] (Unit: mol/L) and the other parameters of the simulation are listed in Table 1.

VISA^+/+^ MEF cells were infected with VSV*GFP and the culture supernatants were collected after 8, 12, 16, 24 and 36 hrs of incubation, respectively. Experimental data showed that virus concentration in the culture supernatants first increased and then decreased at the indicated times ant reached to a peak at 24 hrs p.i. (Figure 3(A)). Based on the real biological meanings of the three components and reaction rates, we determined the best group of parameters in the model. The parameter values of the model and the initial values used for the simulation are listed in the fifth column in Table 1 and Table 2, respectively. Figure 3 shows the consistency between the numerical simulation and the experimental results, indicating that the model is reasonable and can reflect known biological phenomena.

**Table 2:** Initial concentration values of three components for the simulation in Figure 3.

Component	Initial Value	Unit
*V*(0)	100	mol/L
*I*(0)	10	mol/L
*A*(0)	2	mol/L

### Antiviral Immune Responses when there is no Synergistic Effect in the IFN Positive Feedback

**Figure 4:**
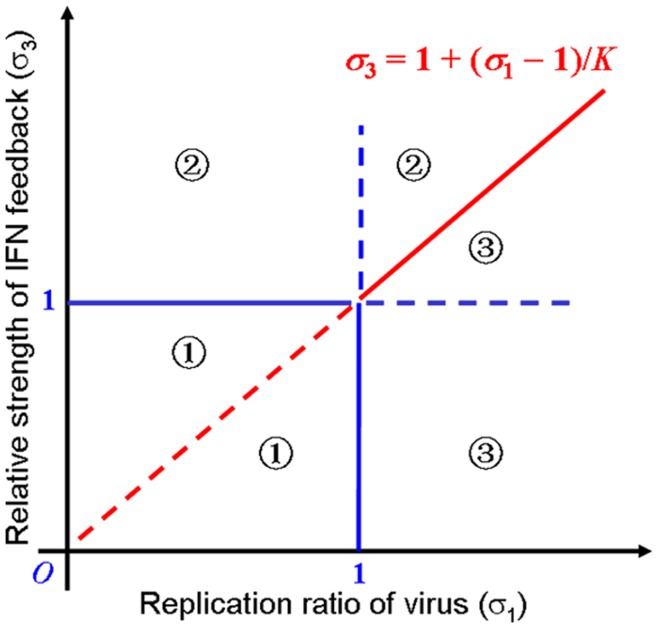
Schematic diagram of stability conditions without a synergistic effect. The first quadrant in the plane *σ*_1_–*σ*_3_ is divided into three regions 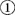, 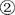 and 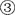 by the lines *σ*_1_ = 1, *σ*_3_ = *1*, and 
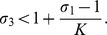

When all reactions are prompt and there is no synergistic effect in the IFN positive feedback, we assume that all time delays are equal to zero and the Hill coefficients *n*_1_ and *n*_2_ are equal to 1. To guarantee the existence of a stable virus-clearance steady state and a stable steady state of virus-latent infection, we performed the stability analysis on the model (see Theorem S1 in the Text S1 for details and the numerical simulation in Figure S1). For convenience to understand, the conditions on the parameters are listed in Table 3. The stability domains can also be represented graphically by three regions that are divided by three straight lines *σ*_1_ = 1, *σ*_3_ = 1, and 
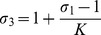
 in the *σ*_1_–*σ*_3_ plane (Figure 4).

**Table 3:** The stability conditions at the steady states for Hill coefficients *n*_1_ = *n*_2_ = 1.

steady-state	Stability conditions	corresponding region in Fig. 4	additional conditions
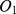	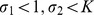	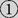	––––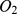	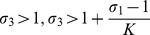	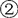	––––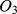	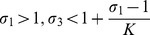	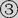	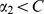

**Figure 5:**
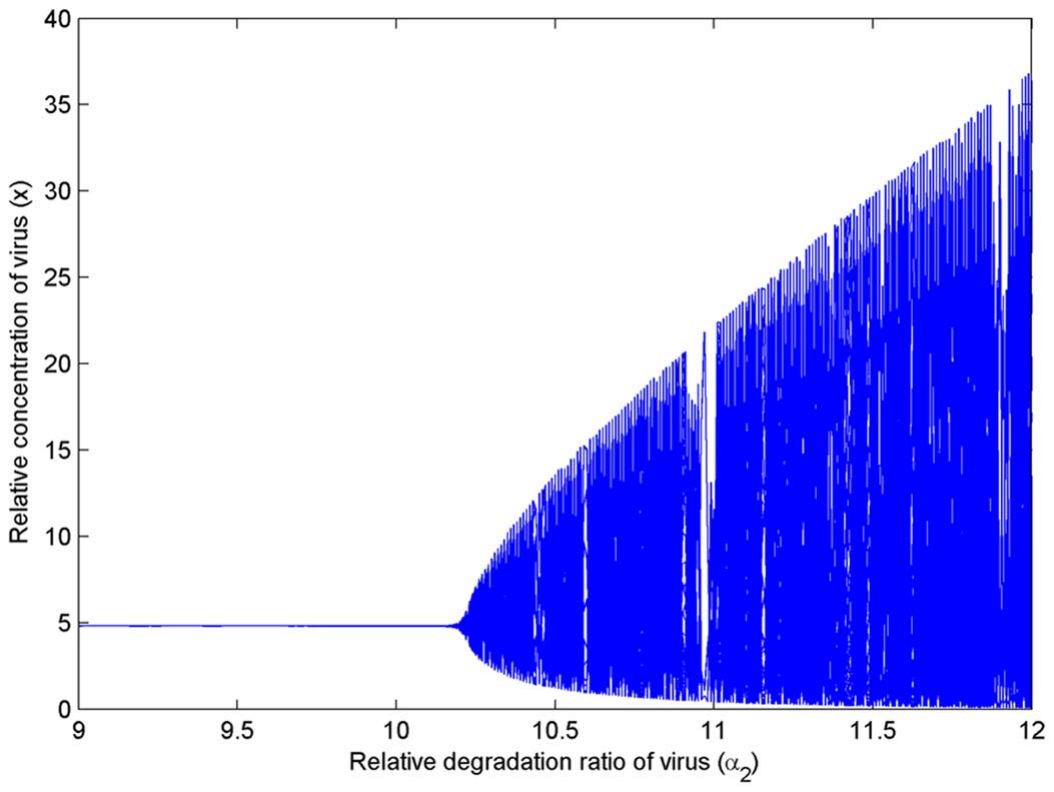
Bifurcation graph about parameter α_2_ without a synergistic effect. The dimensionless parameter α_2_ is associated with the relative ratio of the viral degradation. When *α*_2_ = 10.2347 (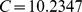), a Hopf bifurcation occurs. The fixed dimensionless parameter values are *n*_1_ = *n*_2_ = 1, *σ*_1_ = 4, *σ*_2_ = 3, *α*_4_ = 4 and *K* = 2.

Remark: 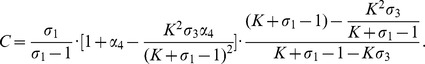

From Table 3 and Figure 4, it can be observed that either the conditions *σ*_1_<1 and *σ*_3_>1 or the conditions *σ*_1_>1 and 
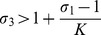
 guarantee the existence of a stable virus-clearance state. This result shows that the virus can be steadily eliminated by immune defenses if the relative strength of the IFNs exceeds a certain threshold value. Thus, viral infection can be eradicated from the host as long as the production of the IFNs is sufficient. If the relative strength of the IFNs is below this threshold (that is, 
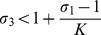
), the system can exhibit two cases. One case is a trivial steady state if *σ*_1_<1, indicating the absence of all components. In the other case, the system exhibits a stable virus-latent state if *σ*_1_>1 and the relative degradation ratio of virus *α*_2_ does not exceed a certain threshold value (*C*) (A special case is presented in Corollary S1 in Text S1. In this case, the relative degradation rate of virus 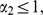 that is, the rate of viral degradation is less than that of AVPs). This result indicates that the infectious viruses are steadily coexisting with the host when the viral reproduction and the activation of IFNs are under controlled states. Otherwise, when the degradation rate of viral mRNAs is large (especially, the degradation of viral mRNAs is faster than that of IFNs), the system will become unstable (undergo oscillation) (see Figure 5 for the bifurcation graph and Theorem S2 for the proof in Text S1).

### Antiviral Immune Responses when there is a Synergistic Effect in the IFN Positive Feedback

**Figure 6:**
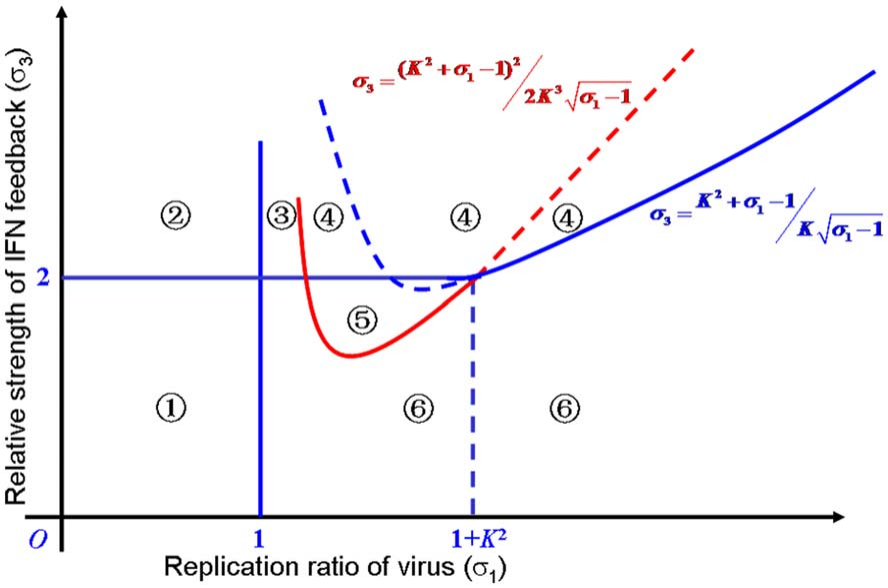
Schematic diagram of stability conditions with a synergistic effect. The first quadrant in the plane *σ*_1–_ *σ*_3_ is divided into six regions 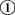, 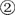, 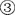, 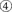, 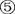 and 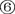 by the lines *σ*_1_ = 1, *σ*_3_ = 2, and the curves 
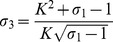
 and 
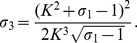

When there is a synergistic effect in the IFN positive feedback, the Hill coefficients *n*_1_ and *n*_2_ are set to be greater than one. Table 4 lists the stability conditions for three steady states and Figure 6 depicts the stability domains (see Theorem S3 and its proof in Text S1 and the numerical simulation in Figure S2).

**Table 4:** The stability conditions at the steady states for Hill coefficients *n*_1_ = *n*_2_ = 2.

steady-states	stability conditions	corresponding region in Fig. 6	additional conditions
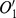	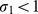	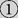 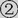	––––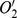	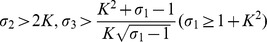	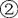 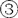 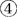	––––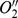	––––	––––	––––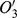	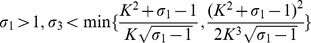	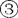 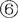	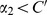

**Figure 7:**
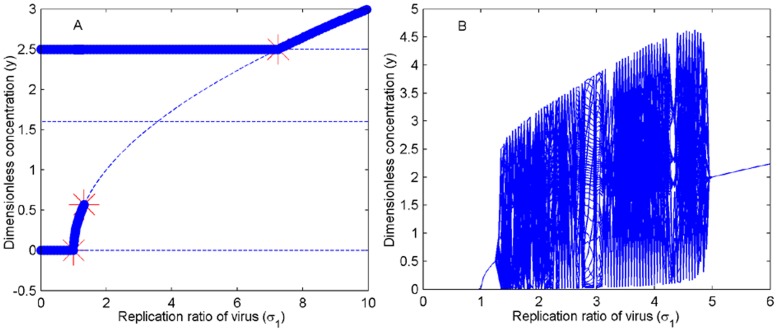
Bistability and bifurcation phenomena. (A). Bistability occurs when the value of *σ*_1_ changes from 0 to 10, which successively pass through domains 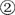, 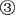, 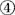 and 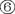 (*σ*_2_ = 4.1) in Figure 6. (B). Bifurcation occurs when the value of *σ*_1_ changes from 0 to 6, which successively pass through domains 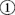, 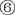, 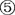 and 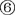 (*σ*_2_ = 3.9) in Figure 6. The other parameters are fixed: *α*_2_ = 0.5, *α*_4_ = 4, *K* = 2 and *n*_1_ = *n*_2_ = 2.

Remark: 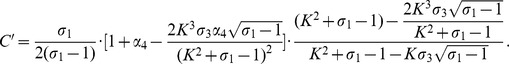

As observed in Table 4 and Figure 6, the system can reach two stable steady states, including the trivial steady state and the virus-clearance steady state, if the replication ratio of the virus *σ*_1_<1 and the relative strength of the IFN positive feedback *σ*_3_>2 (domain 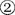 in Figure 6 and Figure 7(A)). We easily infer that the system is also bistable between the virus-clearance steady state and the virus-latent steady state (domain 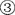 in Figure 6 and Figure 7(A)) when the conditions 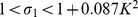 and σ_3_>2 are satisfied, *i.e.*, the system can either be in a virus-clearance state or in the virus-latent state depending on its history (or initial conditions). Conditions *σ*_1_>1 and 
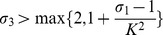
 guarantee the maintenance of a stable virus-clearance state. These results indicate that the IFN positive feedback makes the system exhibit more complicated phenomenon.

Figure 7(A) shows that the system (2) becomes bistable at *O*_1_′ or *O*_2_′ when *σ*_1_ ranges from 0 to 1 and then at *O*_2_′ or *O*_3_′ when *σ*_1_ ranges from 1 to 1.3253. The system (2) becomes monostable at *O*_2_′ when *σ*_1_ ranges from 1.3253 to 7.25 or at *O*_3_′ when *σ*_1_>7.25. Figure 7(B) shows that a Hopf bifurcation occurs when the value of *σ*_1_ changes from 0 to 6, which will successively pass through domains 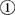, 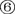, 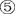 and 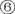 in Figure 6 in turn. The bifurcation graph of α_2_ and the simulation results under different parameter settings are depicted in Figure S3 and Figure S4, respectively. Therefore, the system (2) exhibits characteristics of switches from bistability, to monostability to oscillation and then back to monostability.

### Experimental Verification of the Dynamic Characterization of Virus Replication in Infected Cells

**Figure 8:**
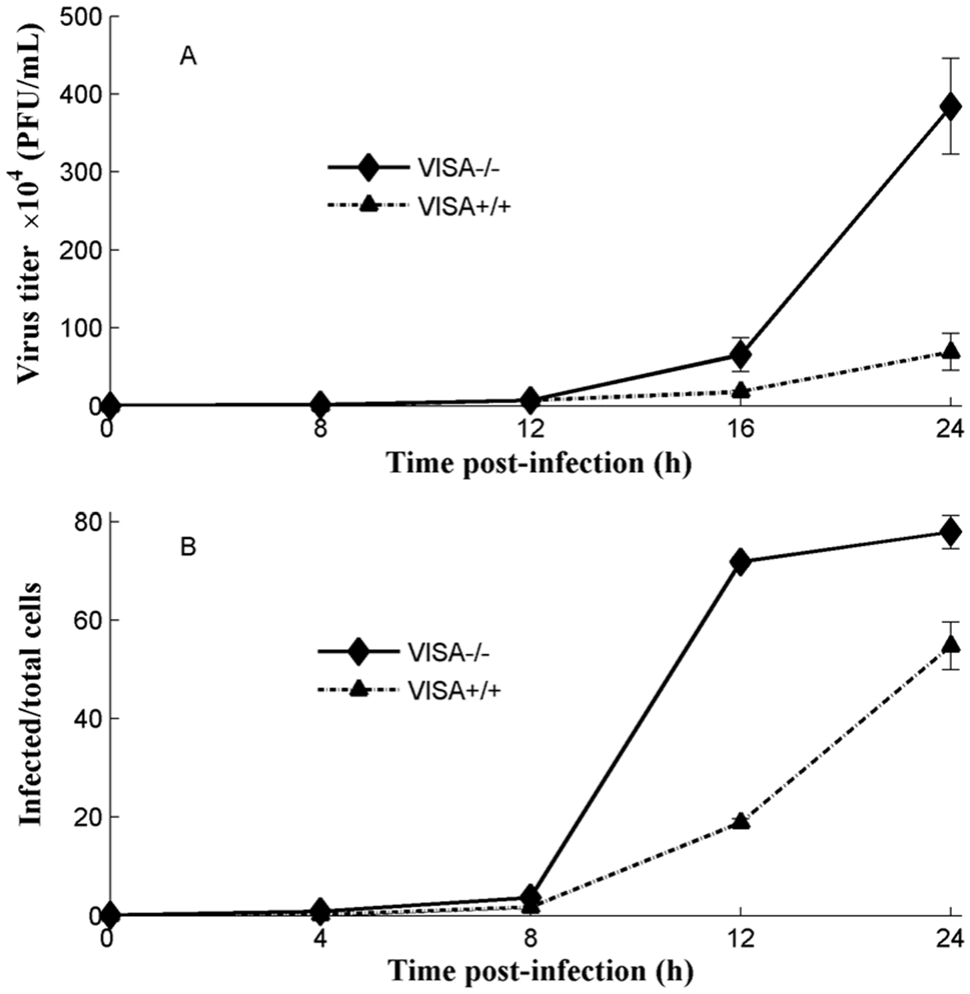
The effect of IFNβ on virus replication. (A). Virus titers in IFNβ-producing (VISA^+/+^) and IFNβ-non-producing (VISA^−/−^) cells were determined by the plaque assay at the indicated times. The data represent the mean ± SD of 3 independent tests. (B). Virus titers were detected using a FACS assay. IFNβ-producing (VISA^+/+^) and IFNβ-non-producing (VISA^−/−^) cells were infected with 0.05 MOI of VSV*GFP and virus-infected populations were determined by negatively gating on uninfected samples at the indicated times. The virus-infected to total cells ratio (% virus positive cells) of VISA^+/+^ or VISA^−/−^ cells are shown. The data represent the mean ± SD of 3 independent tests.

**Figure 9:**
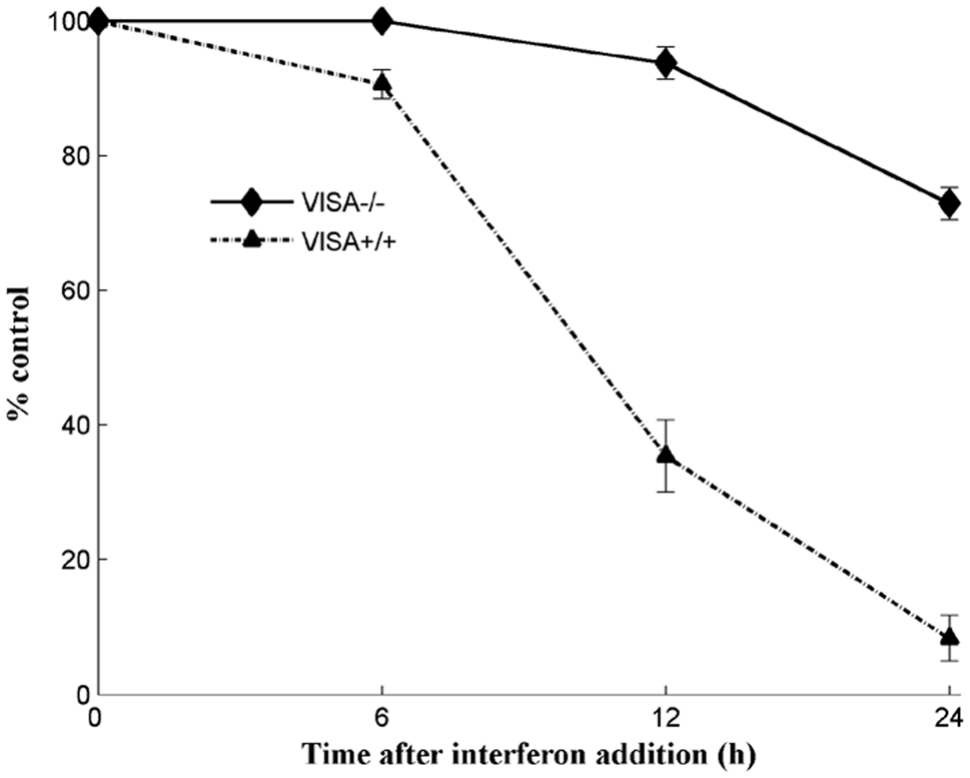
The kinetics of IFNβ-mediated inhibition of virus infection. After 1×10^4^ IFNβ-producing (VISA^+/+^) cells in each well (96-well plates) were treated with the supernatant containing IFNβ for the indicated times, the cells were infected with 0.01 MOI of VSV*GFP. After 24 hrs of incubation, the virus-infected wells were calculated under a fluorescence microscope. The control represents the number of virus-infected wells at the control condition (untreated with the supernatant containing IFNβ).

Silencing VISA significantly decreased IFNβ production and increased virus titers in infected cells [34], [35]. Therefore, wt VISA^+/+^ and VISA^−/−^MEF cells were used to evaluate the effect of IFNβ on the viral replication. At the indicated times p.i.with VSV*GFP, virus titers were determined. From 0 to 8 hrs p.i., low virus titers were observed in both VISA^+/+^ and VISA^−/−^ cells. Subsequently, virus titers increased and reached a peak at 24 hrs p.i. in both VISA^+/+^ and VISA^−/−^ cells. Compared with VISA^+/+^ cells, significantly increased virus titers were observed at 16 and 24 hrs p.i. in VISA^−/−^ cells (Figure 8(A)). Similarly, data of a FACS-based assay showed that a virus titer reached a peak at 24 hrs p.i. in both VISA^+/+^ and VISA^−/−^ cells and that compared with VISA^+/+^ cells, a higher virus titer at 12 and 24 hrs p.i. in VISA^−/−^ cells (Figure 8(B)). A FACS-based assay was believed to be an objective and reproducible method for quantification of virus titers [19], [36]. After induction with Sendai virus, a IFNβ production could be measured at 8 hrs p.i. and exhibited subsequently increased concentration at 12 and 24 hrs p.i. in VISA^+/+^ cells. However, the negligible IFNβ was expressed in VISA^−/−^ cells from 8 to 24 hrs p.i. (Figure S5). When VISA^+/+^ cells were treated with culture supernatant (IFNβ) for 6, 12, and 24 hrs, inhibtion of virus infection significantly increased with the extended treatment time. In contrast, this phenomena was not observed in VISA^−/−^ cells (Figure 9). These results suggest that IFNβ-mediated inhibition of viral replication is dose-dependent and that the inhibition of virus replication is enhanced by IFNβ positive feedback expression.

### Consideration of the Time Delays Under Normal Response Conditions

**Figure 10:**
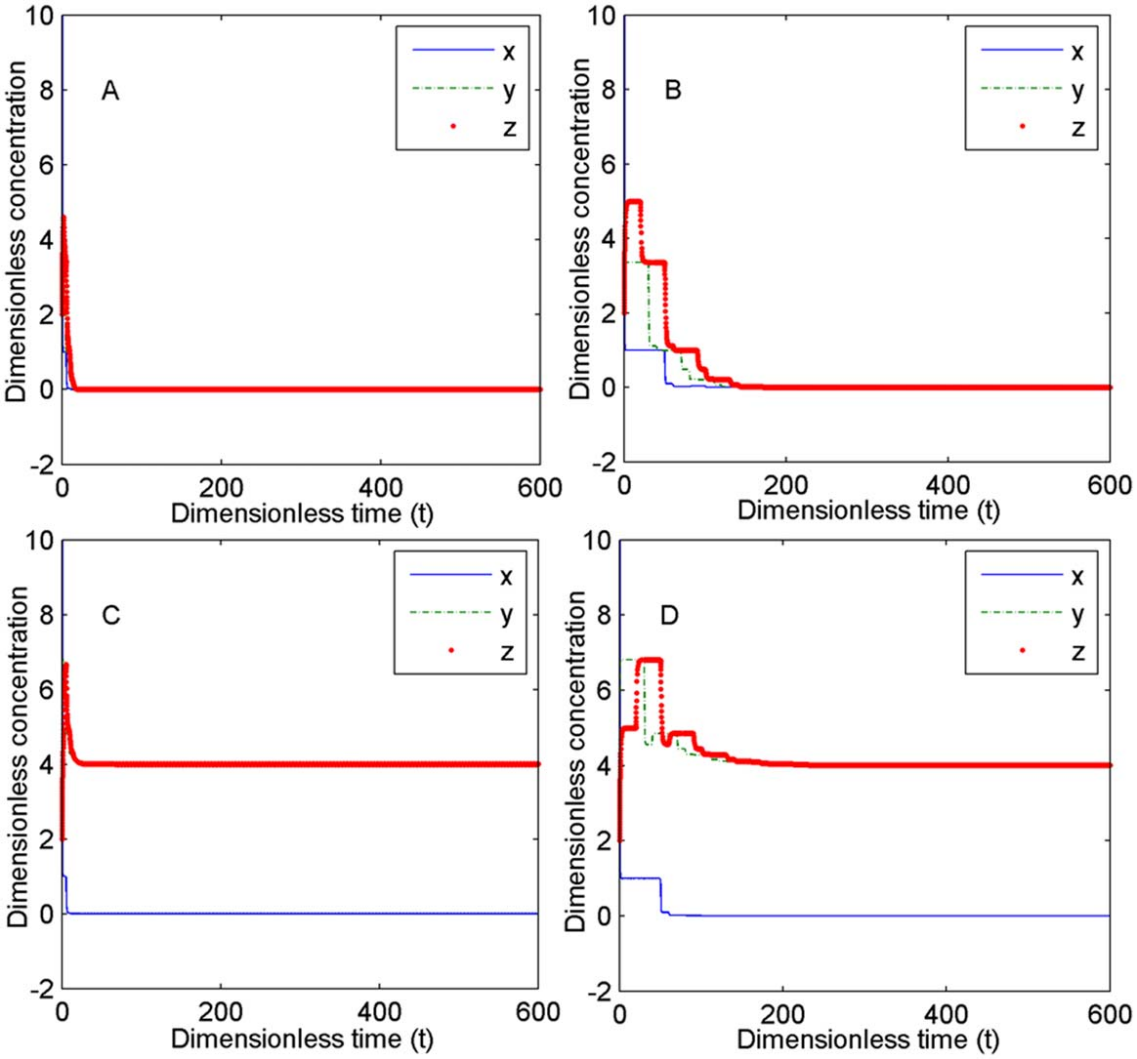
The influence of time delays on the stability of the system. The settings of the dimensionless parameters are *n*_1_ = *n*_2_ = 2, *σ*_1_ = 0.5, *α*_2_ = 5, *α*_4_ = 4, *K* = 2, and the initial value is [10 5 2] for all. *σ*_2_ = 1 for A and B. *σ*_2_ = 5 for C and D. *τ*_1_ = 5, *τ*_2_ = 3, *τ*_3_ = 2, *τ*_4_ = 4, *τ*_5_ = 6 for A and C, and *τ*_1_ = 50, *τ*_2_ = 30, *τ*_3_ = 20, *τ*_4_ = 40, *τ*_5_ = 60 for B and D.

In the simplified IFN-related pathway, we equivalently used the time delays to represent the multistep reaction processes. The impact of time delays on the system was further investigated. For convenience in the theoretical analysis, we assume that the Hill coefficients *n*_1_ = *n*_2_ = 1. We theoretically prove that the time delays have no influence on the stability of the system in the first and second steady states (see Theorem S4 and Theorem S5 for the proofs in Text S1). These theoretical results show that when the replication ratio of the virus (*σ*_1_) is smaller than one, the virus can be eliminated by the immune defense regardless of how the time delays are changed, indicating that the speed (fast or slow) of the chemical reaction processes has no influence on the clearance of virus. Furthermore, we simulate the case in which the Hill coefficients *n*_1_ and *n*_2_ are greater than one and also find that the time delays have no effects on the stability in the first and second steady states (Figure 10).

We further determine the influence of the time delays on the third, virus-latent steady state and find that the delays in different reaction processes have different influences on the system.

### The Influence of Viral Replication and IFN Positive Feedback on the Dynamics of the System

**Figure 11:**
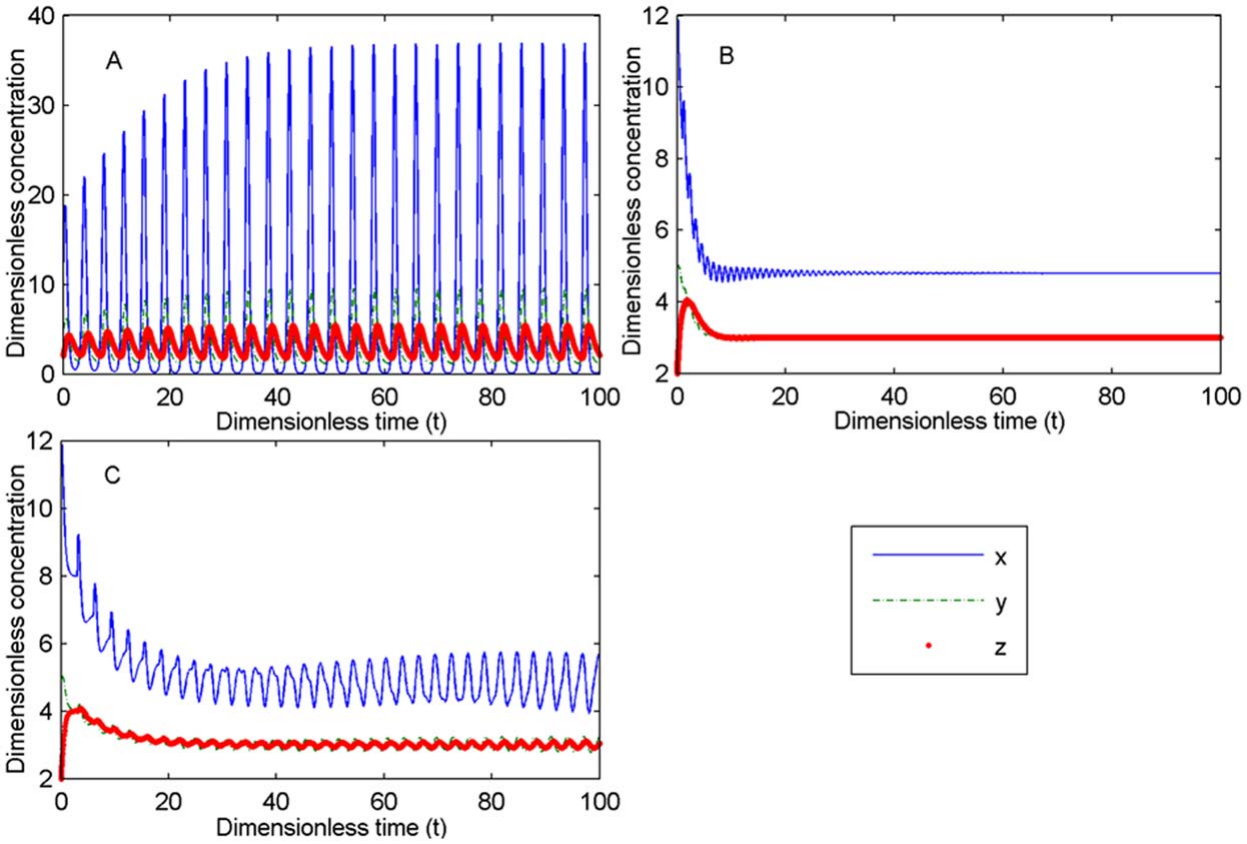
Stabilized process of the oscillation system with time delay τ_1_. (A). *τ*_1_ = 0, oscillation system. (B). *τ*_1_ = 1, steady state. (C). *τ*_1_ = 3, oscillation system. Parameters: *n*_1_ = *n*_2_ = 1, *σ*_1_ = 4, *σ*_2_ = 3, *α*_2_ = 12 (>C = 10.2347), *α*_4_ = 4, *K* = 2. All other time delays are 0.

**Figure 12:**
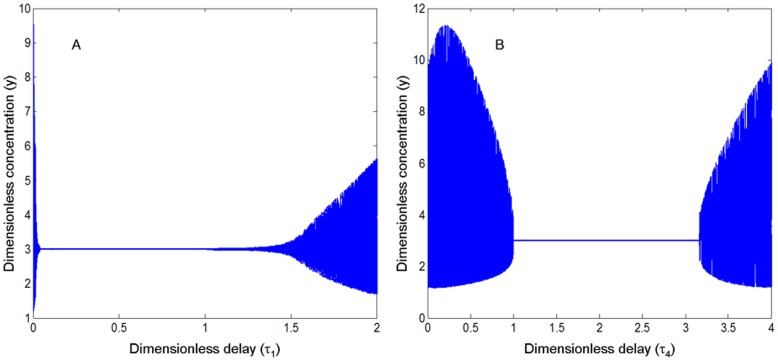
Bifurcation diagram about time delays τ_1_ and τ_4_. (A). System (2) undergoes a process from oscillation to stability and then to the oscillation again when *τ*_1_ changes from 0 to 2. (B). System (2) undergoes a process from oscillation to stability and then to oscillation again when *τ*_4_ changes from 0 to 4. The dimensionless parameters are *n*_1_ = *n*_2_ = 1, *σ*_1_ = 4, *σ*_2_ = 3, *α*_2_ = 12 (>C = 10.2347), *α*_4_ = 4 and *K* = 2. All other time delays are 0.

All time delays can make the system oscillate in the third, virus-latent steady state and induce Hopf bifurcation (see Theorem S6 for the proof in Text S1). However, the effect of the duration of viral replication *τ*_1_ and the time for IFN positive feedback *τ*_4_ on the system is notable. Figure 11 illustrates the process, which moves from an oscillation system when there are no time delays (Figure 11(A)) to a steady state when there is a small delay *τ*_1_ (Figure 11(B)) and then back to an oscillation system when there is a large delay *τ*_1_ (Figure 11(C)). An appropriate range of the time of viral replication and the IFN feedback makes the system stable. The bifurcation graph about *τ*_1_ and *τ*_4_ is given in Figure 12, indicating that the system is stable and infection can be control when the duration for virus replication and IFN positive feedback is within certain range, or the system is unstable (oscillation) and infection would be sustained. From the bifurcation graph, we can also estimate that the time ranges of virus replication and the IFN feedback in innate immune system are approximately 2–10 hrs and 9–25 hrs (dimensionless time range multiply the rate of degradation of the AVPs), respectively.

**Figure 13:**
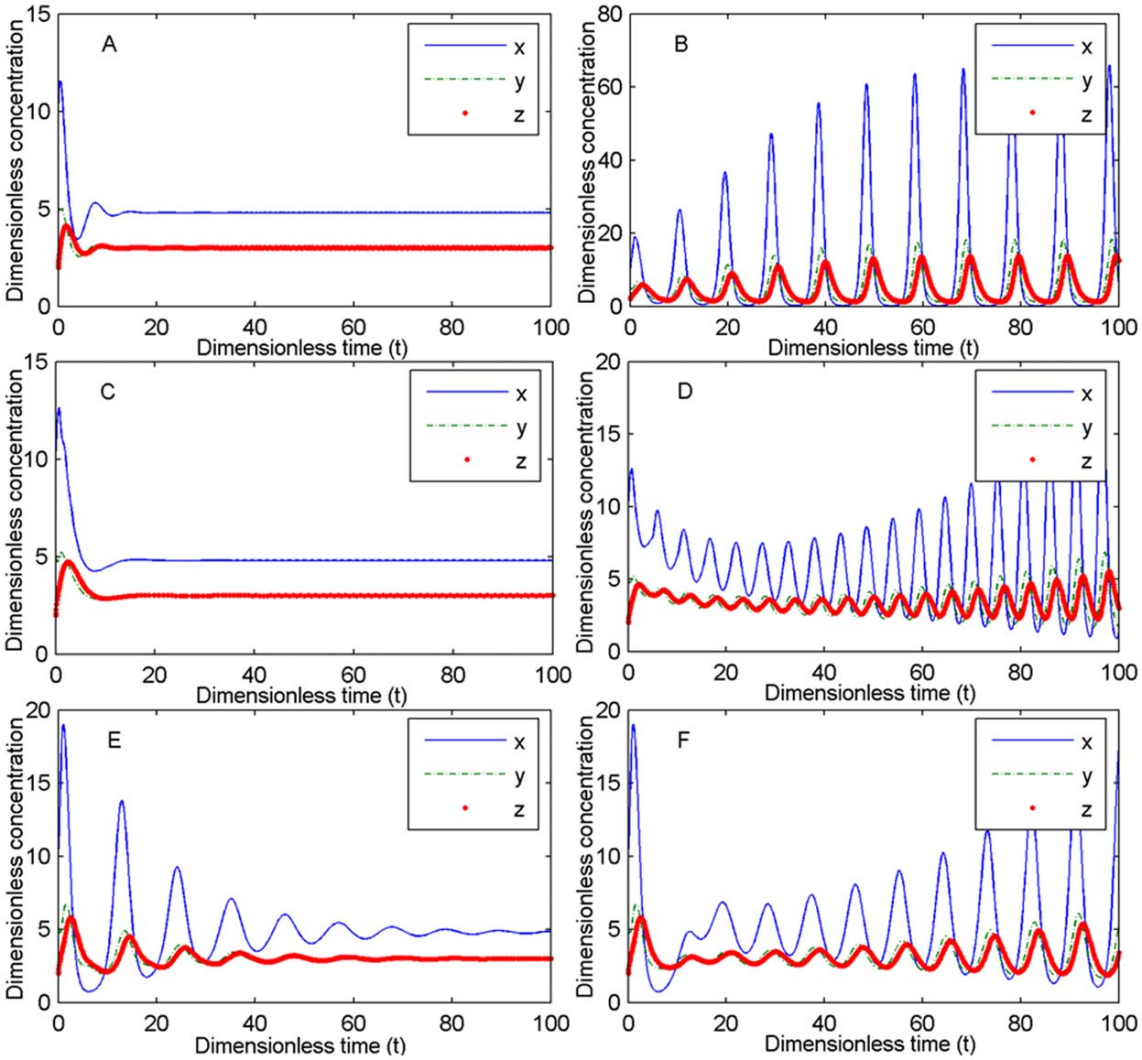
From oscillation caused by the time delays to stability from the time delay τ_1_. (A). System (2) is stable when *τ*_1_ = *τ*_2_ = *τ*_3_ = *τ*_4_ = *τ*_5_ = 0. (B). System (2) is oscillating, induced by *τ*_2_, *τ*_3_ or *τ*_5_ when *τ*_1_ = 0, *τ*_2_ = 0.3, *τ*_3_ = 0.2, *τ*_4_ = 0 and *τ*_5_ = 0.6. (C). System (2) is stable when *τ*_1_ = 1, *τ*_2_ = 0.3, *τ*_3_ = 0.2, *τ*_4_ = 0 and *τ*_5_ = 0.6. (D). System (2) is oscillating when *τ*_1_ = 5, *τ*_2_ = 0.3, *τ*_3_ = 0.2, *τ*_4_ = 0 and *τ*_5_ = 0.6. (E). System (2) is stable when *τ*_1_ = 0, *τ*_2_ = 0.3, *τ*_3_ = 0.2, *τ*_4_ = 4 and *τ*_5_ = 0.6. (F). System (2) is oscillating when *τ*_1_ = 0, *τ*_2_ = 0.3, *τ*_3_ = 0.2, *τ*_4_ = 9 and *τ*_5_ = 0.6. Other parameters: *n*_1_ = *n*_2_ = 1, *σ*_1_ = 4, *σ*_2_ = 3, *α*_2_ = 2.5 (<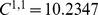), *α*_4_ = 4 and *K* = 2. The initial values are [10,5,2].

**Figure 14:**
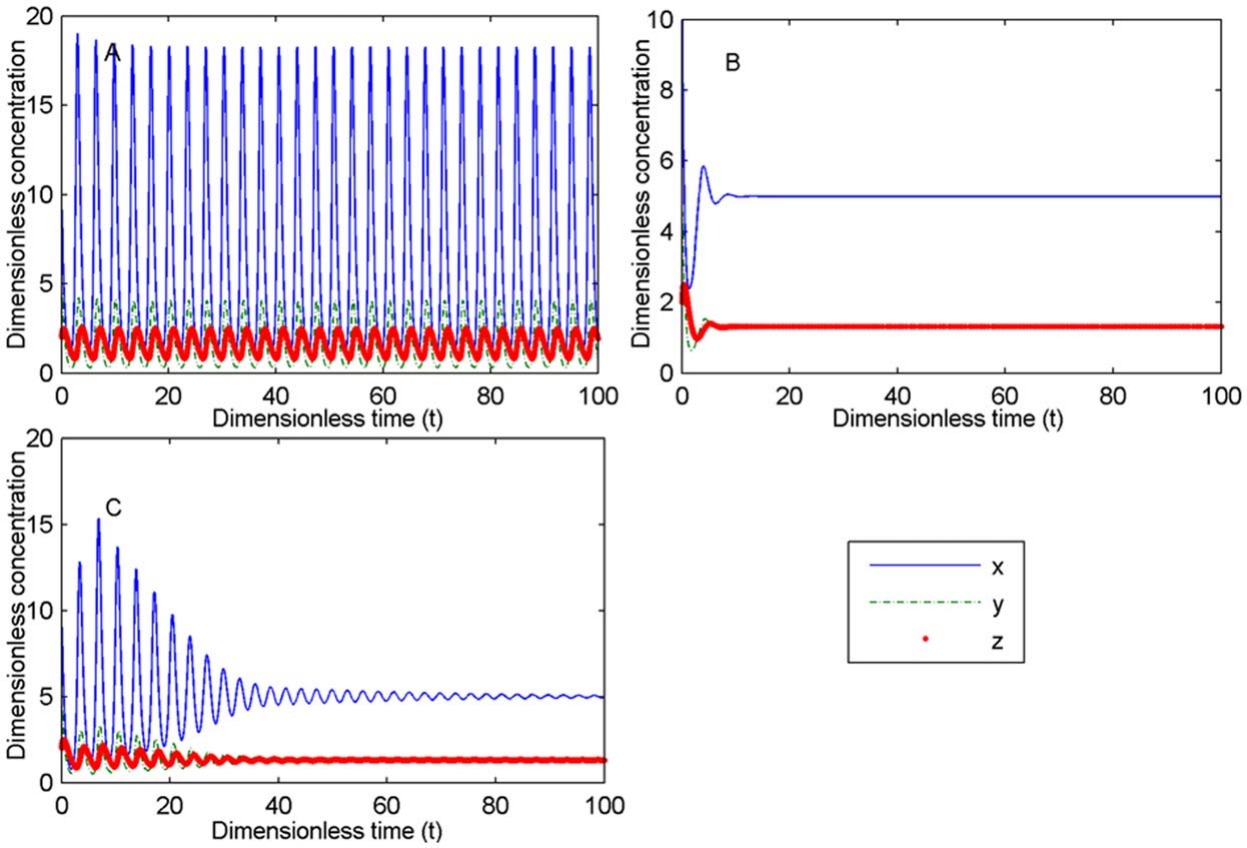
Stabilization of oscillation when Hill coefficients n_1_ and n_2_ are greater than one. (A). No delays, oscillation system. (B). *τ*_1_ = 1, steady state. (C). *τ*_4_ = 5, steady state. Other dimensionless parameters: *n*_1_ = 4, *n*_2_ = 3, *σ*_1_ = 4, *σ*_2_ = 0.3, *α*_2_ = 2, *α*_4_ = 4 and *K* = 2. The initial values are (10, 5, 2).

Moreover, *τ*_1_ and *τ*_4_ can eliminate the oscillation caused by other three time delays *τ*_2_, *τ*_3_ and*τ*_5_ in three reactions ((2), (3) and (4)) in Figure 2 (Figure 13). Figure 13 indicates that the system is capable of changing from a steady state with no delays (Figure 13(A)) to oscillation with delays *τ*_2_, *τ*_3_ and *τ*_5_ (Figure 13(B)) and then to a steady state with a small delay *τ*_1_ (Figure 13(C)) and finally back again to oscillation with a large delay *τ*_1_ (Figure 13(D)). The time of the IFN feedback regulation *τ*_4_ has similar results as *τ*_1_ (Figure 13 (E) and (F)). When *n*_1_ and *n*_2_ are greater than 1, a large delay *τ*_1_ or *τ*_4_ can also make the system stable (Figure 14). These results demonstrated that an adaptable time delay for viral replication and IFN feedback regulation will control the dynamics of the system even if the delays of the other reaction processes affect the innate immune system, further suggesting the importance of two processes for viral replication and IFN feedback regulation.

## Discussion

Understanding the dynamic regulation of antiviral immune responses is essential for predicting the manifestation and outcome of infectious disease. Unfortunately, as it relates to complex biological processes, the acquisition of the empirical data is usually time-consuming and intricate and is tremendously limited by both temporal and financial constraints. In recent years, computational modeling and analysis based on experimental measurements have become very important tools for predicting the intrinsic mechanisms of signaling pathways at the network level [37]. However, data-driven models of signaling networks are often complicated because there are many constant and adjustable parameters in the models, which include rate and equilibrium constants together with initial species concentrations [38], [39]. A relatively realistic method to address the problem is to identify and model only the essential feature(s) of each process in the networks [37].

The present model only considers the three important components in virus-triggered IFN signaling pathways. Thus, it is beneficial to analyze the model using dynamic theory. We theoretically obtained the quantitative relationship between the parameters in the model and analyzed the effects of model parameters and feedback mechanisms on the behaviors of the system after virus infection. Therefore, it is quite different from the models in the previous studies. In previously described models, only specific parameter sets are numerically simulated and it is not convenient to determine the influence of varying values of these parameters. The mathematical model we developed in this study will enable investigators to gain a better understanding of the virus-triggered innate immune signaling network.

To verify our theoretical results, IFNβ-producing (VISA^+/+^) and IFNβ-non-producing (VISA^−/−^) cells were used to investigate the dynamics of virus-induced IFN production and its antiviral response. The virus titers were directly calculated by measuring cells infected with a VSV*GFP using fluorescence microscopy and a flow cytometer. The virus titers in cells were tested at 0, 8, 12, 24, 36, 48 and 72 hrs p.i., respectively. Our results showed that the virus titer reached a peak at 24 hrs p.i. and subsequently decreased. Compared with VISA^+/+^ cells, 9-fold higher peak viral titers were produced in VISA^−/−^ cells. Because a FACS-based assay is used to detect infected cells (not for mature virus), a higher virus titer was obtained than TCID_50_ at 12 hrs p.i. After treatment with the culture supernatants containing IFNβ for 6, 12 and 24 hrs, respectively, cells were infected with VSV*GFP, and inhibition of the virus infection was enhanced following a prolonged treatment in VISA^+/+^ cells, but not in VISA^−/−^ cells, suggesting that inhibition of virus replication by IFNβ was a dose-dependent manner. However, the real innate immune response processes of a host are complicated; therefore, in addition to integrating experimental studies with mathematical models, a suitable animal model will be developed to better understand the mechanisms of virus-triggered immune responses and viral diseases.

Previous studies indicated that the time delay is a key factor in the behavior of the model and that the steady state of the system is destabilized if the time delay is long enough [40–44]. In many literatures related to biological systems, a single time delay was considered because multiple time delays make the model complex and the resulting theoretical analysis difficult [45]. In this study, we consider five time delays in reaction processes. By combining the theoretical proofs with numerical simulations, we find that appropriate time delays could stabilize the system and switch it from an unstable or oscillating condition to a steady state. It appears as if time delays in innate immune responses could be beneficial in lessening the pathological damage induced by virus infection. This interesting phenomenon has not been previously described in the literature. This helps us understand more properties and regimes of the antiviral innate immune responses. So far, it is difficult to verify the effect of time delay on innate immune responses using biological experiments. The role of time delays in the system based on this model requires further exploration.

In addition, if the specific biological background used in this model is neglected, the present model can be viewed as a coupled system consisting of negative and positive feedback. Importantly, the system can switch between monostability, bistability and oscillation. The specific features of the system could be used to design a reasonable biological network and specify the corresponding biological functions.

### Conclusion

In this study, we propose a simplified mathematical model with time delays for understanding the virus-triggered innate immune network. We deduce that the system can switch between monostability, bistability and oscillation with different conditions. By combining the theoretical analysis with experiment study, we find that the replication ratio of the virus is an important parameter for resulting in viral infection, virus-clearance and steady-state maintenance. If the replication ratio of the virus is less than one, then the virus is eliminated by the immune defense; and if the replication ratio of the virus is relatively high, then the stability of the system is disturbed. In this case, the system either eliminates the virus via IFN positive feedback and AVP production or causes an infectious disease. Moreover, the influence of time delays on the stability of the system depends on various steady-states. The duration of two processes for viral replication and IFN feedback can guarantee the normal innate immune response, beyond this range, the immune balance possibly is disturbed. The predictions in this study can be generalized to other viral infections. Our work indicates that modeling and dynamical analysis of virus-triggered innate immune responses have the potential to prevent excessive experimentation and to provide researchers with complementary and valuable insight into the mechanisms of signaling networks.

## Supporting Information

Figure S1
**Simulation of steady states and bifurcation for the system (2) without a synergistic effect.** (A): System (2) at *O*_1_(0, 0, 0) is stable. The parameters are *σ*_1_ = 0.5, *σ*_2_ = 1, *α*_2_ = 5, *α*_4_ = 4 and *K* = 2, which occur in region 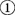 of Figure 4. (B): System (2) at *O*_2_ (0, 1, 1) is stable. The parameters are *σ*_1_ = 0.5, *σ*_2_ = 3, *α*_2_ = 5, *α*_4_ = 4 and *K* = 2, which occur in region 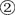 of Figure 4. (C): System (2) at *O*_3_ (4.8, 3, 3) is stable. The parameters are *σ*_1_ = 4, *σ*_2_ = 3, *α*_2_ = 5 (C = 10.2347), *α*_4_ = 4 and *K* = 2, which occur in region 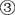 of Figure 4. (D) and (E): Hopf bifurcation phenomenon. At the same time, the system (2) at *O*_1_ (0, 0, 0), *O*_2_ (0, 1, 1) or *O*_3_ (4.8, 3, 3) is unstable when *α*_2_ = 10.2347 for (D) and *α*_2_ = 12 for (E). The other parameters are same: *σ*_1_ = 4, *σ*_2_ = 3, *α*_4_ = 4 and *K* = 2, which occur in region 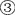 of Figure 4 but *α*_2_> = *C* does not satisfy the additional conditions. Thus a Hopf bifurcation occurs and a periodic oscillation appears. The amplitude of the periodic oscillation is greater if *α*_2_ is larger. The initial values are [10], [5], [2] and *n*_1_ = *n*_2_ = 1 for all simulations.(TIF)

Figure S2
**Simulation of steady states and bifurcation for the system (2) with a synergistic effect.** (A) and (B): The origin is stable. (A): The parameters of *σ*_1_ and *σ*_2_ are in region 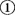 of Figure 4 (*σ*_2_ = 1, *n*_1_ = *n*_2_ = 1, cf. Figure S1: A). B: The parameters of *σ*_1_ and *σ*_2_ are in region 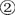 of Figure 4, *i.e*., region 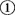 of Figure 6 (*σ*_2_ = 3, *n*_1_ = *n*_2_ = 2). The other parameters are fixed *σ*_1_ = 0.5, *α*_2_ = 5, *α*_4_ = 4 and *K* = 2. (C) and (D): Bistability Phenomena. (C): The equilibrium point *O*_2_′(0,4,4) is locally asymptotically stable. (D): The equilibrium point *O*_1_′(0, 0, 0) is also locally asymptotically stable. The other parameters are fixed: *σ*_1_ = 0.5, *σ*_2_ = 5, *α*_2_ = 5, *α*_4_ = 4, *K* = 2 and *n*_1_ = *n*_2_ = 2 (*σ*_1_ and *σ*_2_ are in region 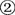 of Figure 6). (E), (F), (G) and (E): System (2) at *O*_3_′(1.7853, 1.7321, 1.7321) is locally asymptotically stable. *α*_2_ = 0.5 and *α*_2_< C′ (C′ = 0.6303). (F) and (G): Hopf bifurcation phenomenon. *α*_2_ = 0.6303 for (F) and *α*_2_ = 5 for (G), but *α*_2_> = C′. The other parameters are fixed: *σ*_1_ = 4, *σ*_2_ = 3, *α*_4_ = 4 and *K* = 2, which are in region 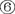 of Figure 6. The initial values are [10], [5], [2] for (A), (B), (C), (E), (F) and (G), and the initial values are [1, 0.5, 0.2] for (D).(TIF)

Figure S3 **Comparison of bifurcation graph about** ***α*** **_2_ without and with a synergistic effect.** (A): When *α*_2_ = 10.2347 (*C = *10.2347, *n*_1_ = *n*_2_ = 1), a Hopf bifurcation occurs (Figure 5 in the main text). (B): When *α*_2_ = 0.6303 (*C′ = *0.6303, *n*_1_ = *n*_2_ = 2), a Hopf bifurcation occurs. The other parameters are same: *σ*_1_ = 4, *σ*_2_ = 3, *α*_4_ = 4 and *K* = 2.
(TIF)

Figure S4
**Bistability and oscillation phenomenon of system (2) with synergistic effect.** (A) and (B): Bistability Phenomena. (A): The equilibrium point *O*_2_′(0, 4, 4) is locally asymptotically stable (the initial values are [10], [5], [2]). (B): The equilibrium point *O*_3_′(0.7771, 0.3162, 0.3162) is locally asymptotically stable (the initial values are [0.1, 0.5, 0.2]). The other parameters are fixed: *σ*_1_ = 1.1, *σ*_2_ = 5, *α*_2_ = 0.5, C′ = 4.41, *α*_4_ = 4, *K* = 2 and *n*_1_ = *n*_2_ = 2 (*σ*_1_ and *σ*_2_ occur in region 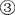 of Figure 6). (C): The equilibrium point *O*_2_′(0,4,4) is locally asymptotically stable (the initial values are [10], [5], [2]). The parameters are *σ*_1_ = 1.5, *σ*_2_ = 5, *α*_2_ = 0.5, *α*_4_ = 4, *K* = 2, and *n*_1_ = *n*_2_ = 2 (*σ*_1_ and *σ*_2_ occur in region 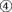 of Figure 6). (D) and (E): Stability blind. *O*_1_′(0,0,0) ((D): the initial values are [10], [5], [2]) and *O*_3_′(1.0951,0.7071,0.7071) ((E): the initial values are [0.1, 0.5, 0.2]) are unstable. The parameters are fixed: *σ*_1_ = 1.5, *σ*_2_ = 3.9, *α*_2_ = 0.5(C′ = −0.2226), *α*_4_ = 4, *K* = 2 and *n*_1_ = *n*_2_ = 2 (*σ*_1_ and *σ*_2_ occur in region 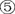 of Figure 6).(TIF)

Figure S5 IFNβ concentration was determined using VSV-based IFN bioassay. IFNβ production in VISA^+/+^ and VISA^−/−^ cells at the indicated induction times by Sendai virus.
(TIF)

Text S1 **This file contains theoretical analysis for the models (1) and (2). (PDF).**
(DOC)
