# Supplementary figures and images for: Modeling and Dynamical Analysis of Virus-Triggered Innate Immune Signaling Pathways
Source: PLoS One. 2012 Oct 30;7(10):e48114. doi: 10.1371/journal.pone.0048114 (PMC3484162; doi:10.1371/journal.pone.0048114)

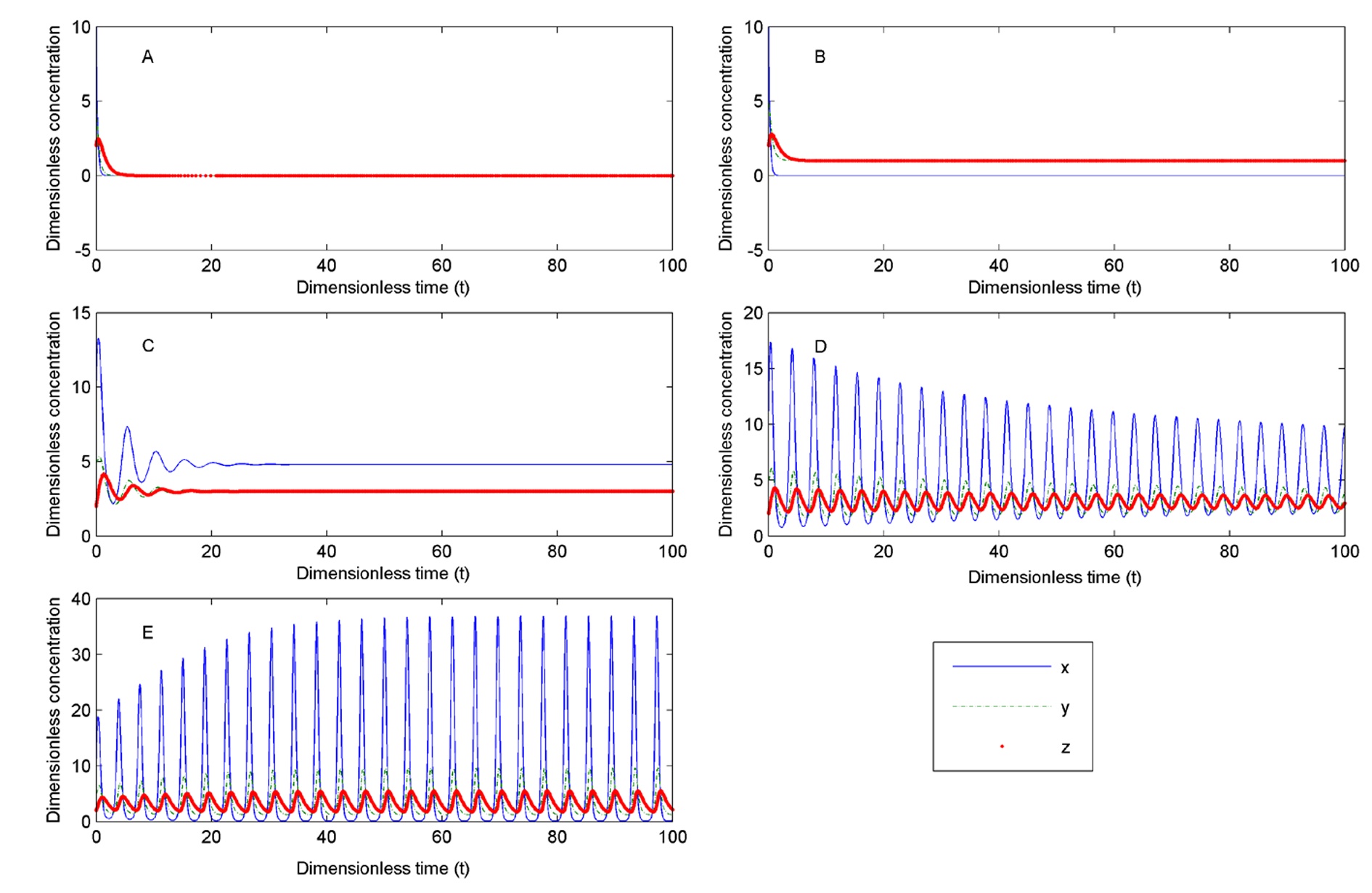

Supplement: Figure S1 — Simulation of steady states and bifurcation for the system (2) without a synergistic effect. (A): System (2) at O 1(0, 0, 0) is stable. The parameters are σ 1 = 0.5, σ 2 = 1, α 2 = 5, α 4 = 4 and K = 2, which occur in region of Figure 4. (B): System (2) at O 2 (0, 1, 1) is stable. The parameters are σ 1 = 0.5, σ 2 = 3, α 2 = 5, α 4 = 4 and K = 2, which occur in region of Figure 4. (C): System (2) at O 3 (4.8, 3, 3) is stable. The parameters are σ 1 = 4, σ 2 = 3, α 2 = 5 (C = 10.2347), α 4 = 4 and K = 2, which occur in region of Figure 4. (D) and (E): Hopf bifurcation phenomenon. At the same time, the system (2) at O 1 (0, 0, 0), O 2 (0, 1, 1) or O 3 (4.8, 3, 3) is unstable when α 2 = 10.2347 for (D) and α 2 = 12 for (E). The other parameters are same: σ 1 = 4, σ 2 = 3, α 4 = 4 and K = 2, which occur in region of Figure 4 but α 2> = C does not satisfy the additional conditions. Thus a Hopf bifurcation occurs and a periodic oscillation appears. The amplitude of the periodic oscillation is greater if α 2 is larger. The initial values are [10], [5], [2] and n 1 = n 2 = 1 for all simulations. (TIF) [file pone.0048114.s001.tif]

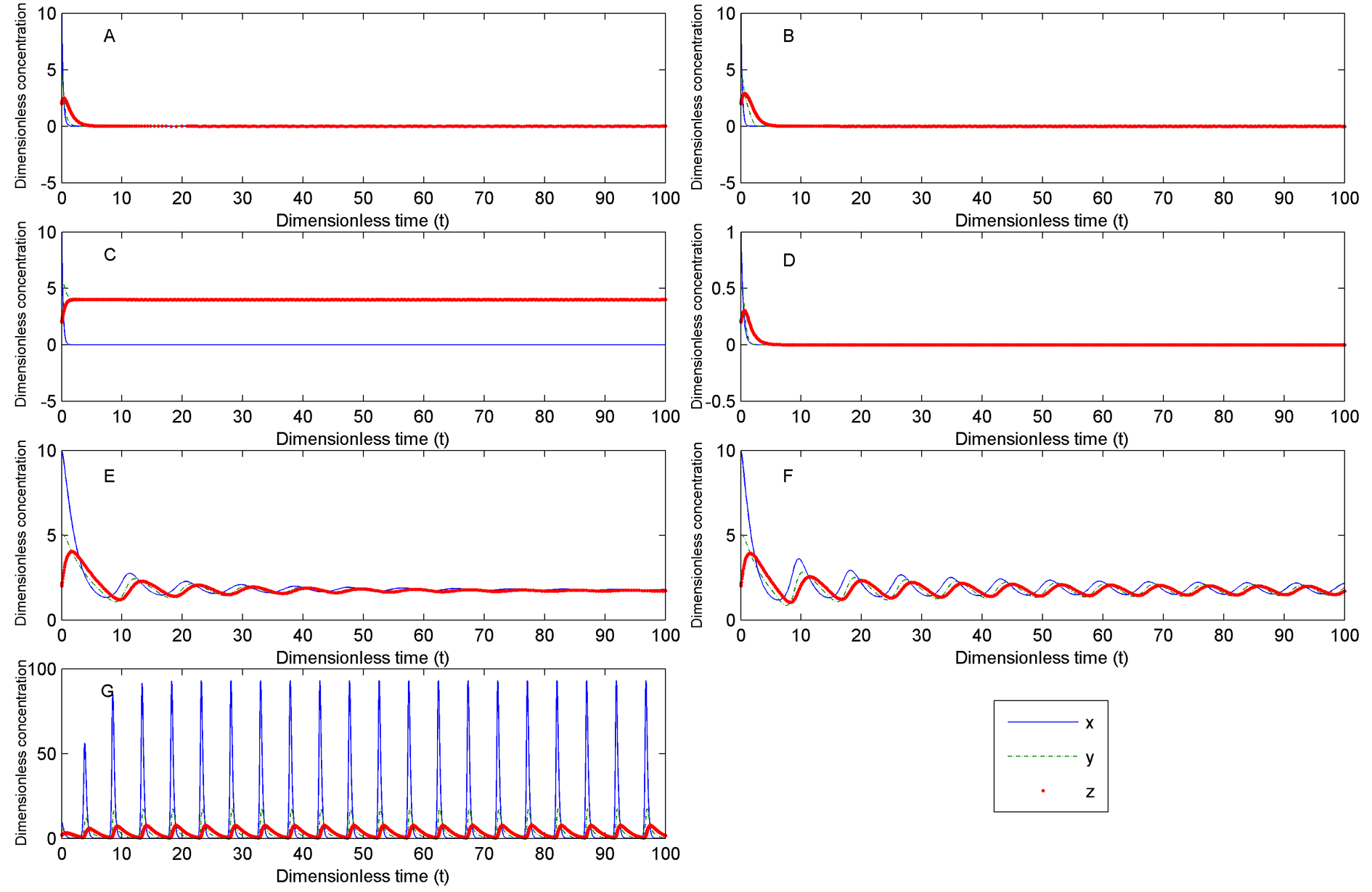

Supplement: Figure S2 — Simulation of steady states and bifurcation for the system (2) with a synergistic effect. (A) and (B): The origin is stable. (A): The parameters of σ 1 and σ 2 are in region of Figure 4 (σ 2 = 1, n 1 = n 2 = 1, cf. Figure S1: A). B: The parameters of σ 1 and σ 2 are in region of Figure 4, i.e., region of Figure 6 (σ 2 = 3, n 1 = n 2 = 2). The other parameters are fixed σ 1 = 0.5, α 2 = 5, α 4 = 4 and K = 2. (C) and (D): Bistability Phenomena. (C): The equilibrium point O 2′(0,4,4) is locally asymptotically stable. (D): The equilibrium point O 1′(0, 0, 0) is also locally asymptotically stable. The other parameters are fixed: σ 1 = 0.5, σ 2 = 5, α 2 = 5, α 4 = 4, K = 2 and n 1 = n 2 = 2 (σ 1 and σ 2 are in region of Figure 6). (E), (F), (G) and (E): System (2) at O 3′(1.7853, 1.7321, 1.7321) is locally asymptotically stable. α 2 = 0.5 and α 2< C′ (C′ = 0.6303). (F) and (G): Hopf bifurcation phenomenon. α 2 = 0.6303 for (F) and α 2 = 5 for (G), but α 2> = C′. The other parameters are fixed: σ 1 = 4, σ 2 = 3, α 4 = 4 and K = 2, which are in region of Figure 6. The initial values are [10], [5], [2] for (A), (B), (C), (E), (F) and (G), and the initial values are [1, 0.5, 0.2] for (D). (TIF) [file pone.0048114.s002.tif]

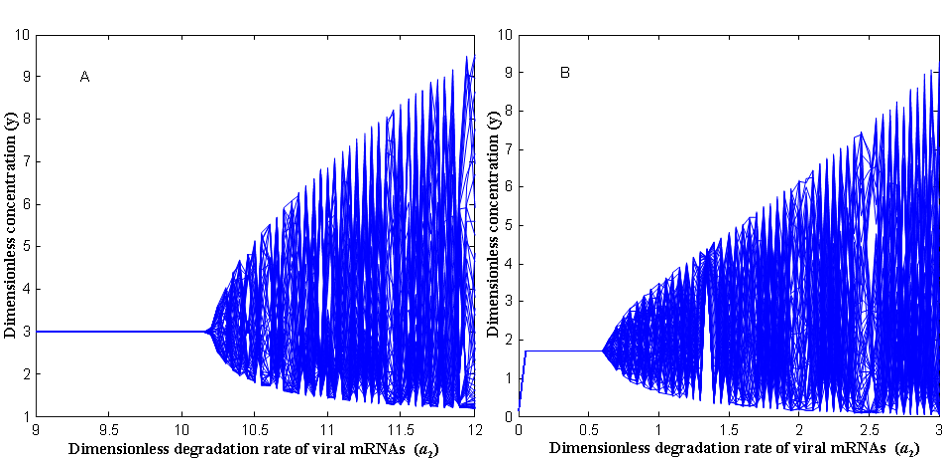

Supplement: Figure S3 — Comparison of bifurcation graph about α 2 without and with a synergistic effect. (A): When α 2 = 10.2347 (C = 10.2347, n 1 = n 2 = 1), a Hopf bifurcation occurs (Figure 5 in the main text). (B): When α 2 = 0.6303 (C′ = 0.6303, n 1 = n 2 = 2), a Hopf bifurcation occurs. The other parameters are same: σ 1 = 4, σ 2 = 3, α 4 = 4 and K = 2. (TIF) [file pone.0048114.s003.tif]

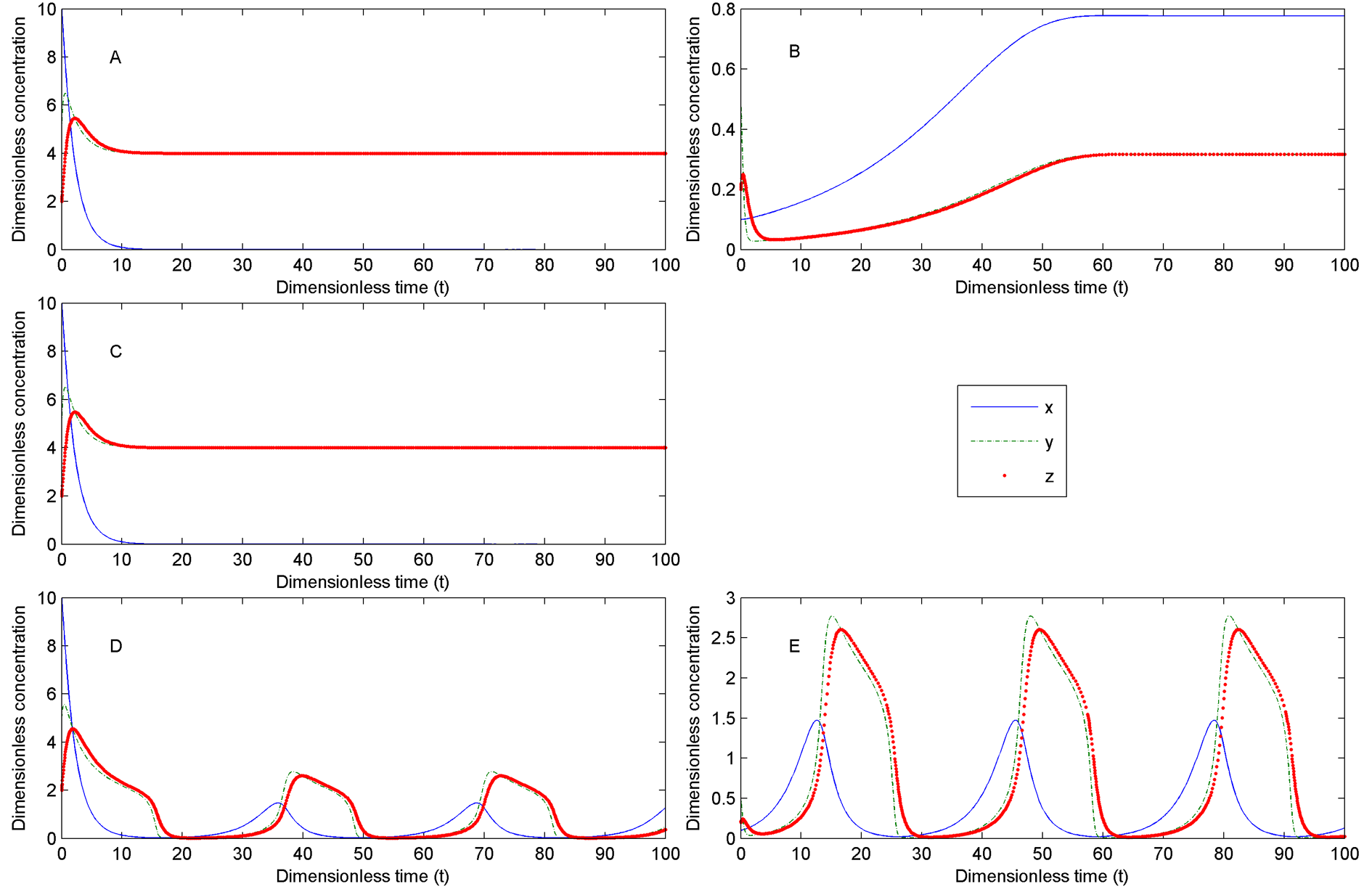

Supplement: Figure S4 — Bistability and oscillation phenomenon of system (2) with synergistic effect. (A) and (B): Bistability Phenomena. (A): The equilibrium point O 2′(0, 4, 4) is locally asymptotically stable (the initial values are [10], [5], [2]). (B): The equilibrium point O 3′(0.7771, 0.3162, 0.3162) is locally asymptotically stable (the initial values are [0.1, 0.5, 0.2]). The other parameters are fixed: σ 1 = 1.1, σ 2 = 5, α 2 = 0.5, C′ = 4.41, α 4 = 4, K = 2 and n 1 = n 2 = 2 (σ 1 and σ 2 occur in region of Figure 6). (C): The equilibrium point O 2′(0,4,4) is locally asymptotically stable (the initial values are [10], [5], [2]). The parameters are σ 1 = 1.5, σ 2 = 5, α 2 = 0.5, α 4 = 4, K = 2, and n 1 = n 2 = 2 (σ 1 and σ 2 occur in region of Figure 6). (D) and (E): Stability blind. O 1′(0,0,0) ((D): the initial values are [10], [5], [2]) and O 3′(1.0951,0.7071,0.7071) ((E): the initial values are [0.1, 0.5, 0.2]) are unstable. The parameters are fixed: σ 1 = 1.5, σ 2 = 3.9, α 2 = 0.5(C′ = −0.2226), α 4 = 4, K = 2 and n 1 = n 2 = 2 (σ 1 and σ 2 occur in region of Figure 6). (TIF) [file pone.0048114.s004.tif]

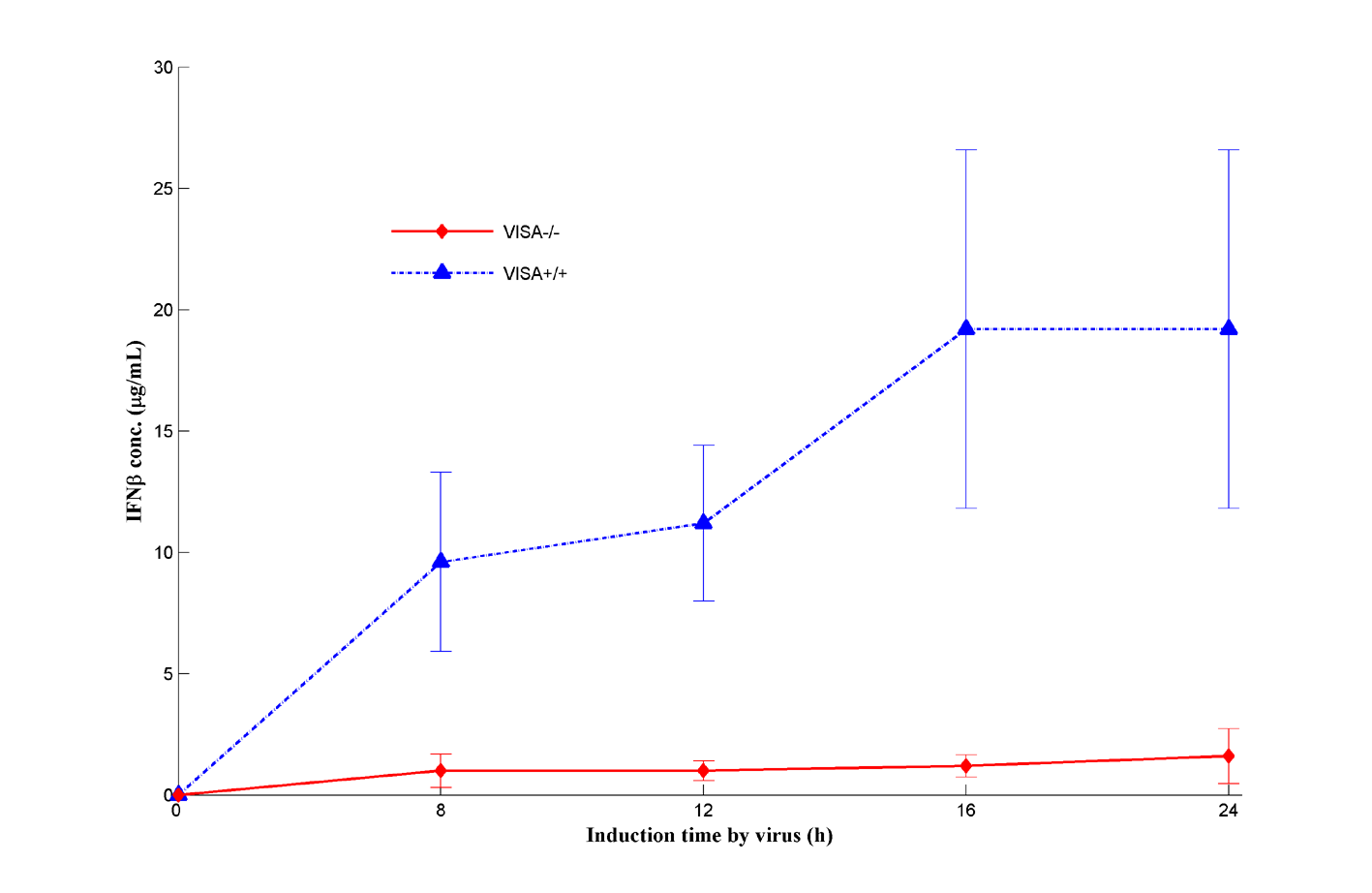

Supplement: Figure S5 — IFNβ concentration was determined using VSV-based IFN bioassay. IFNβ production in VISA+/+ and VISA−/− cells at the indicated induction times by Sendai virus. (TIF) [file pone.0048114.s005.tif]
