# Supplementary material for: Modeling and Dynamical Analysis of Virus-Triggered Innate Immune Signaling Pathways
Source: PLoS One. 2012 Oct 30;7(10):e48114. doi: 10.1371/journal.pone.0048114 (PMC3484162; doi:10.1371/journal.pone.0048114)
Supplement: Text S1 — This file contains theoretical analysis for the models (1) and (2). (PDF). (DOC) [file pone.0048114.s006.doc]

## Modeling and Dynamical Analysis of Virus-triggered Innate Immune Signaling Pathways

## Jinying Tan, Ruangang Pan, Lei Qiao, Xiufen Zou, Zishu Pan

**Theorem S1**

For the model (2) in the main text when $\tau_{i}=0,i=1,2,3,4,5$ and *n*_1_=*n*_2_=1, there are three steady states:

$O_{1}\text{ }(\bar{x}_{1},\bar{y}_{1},\bar{z}_{1})$: $\bar{x}_{1}=\bar{y}_{1}=\bar{z}_{1}=0$

$O_{2}\text{ }(\bar{x}_{2},\bar{y}_{2},\bar{z}_{2})$: $\bar{x}_{2}=0,\text{ }\bar{z}_{2}=\bar{y}_{2}=K(\sigma_{3}-1)$

$O_{3}\text{ }(\bar{x}_{3},\bar{y}_{3},\bar{z}_{3})$: $\bar{x}_{3}=(\sigma_{1}-1)(1-\frac{K\sigma_{3}}{K+\sigma_{1}-1})\alpha_{4},\text{ }\bar{z}_{3}=\bar{y}_{3}=\sigma_{1}-1$

Only one non-negative steady state is locally asymptotically stable under any conditions.

**(1)** If $\sigma_{1}<1$ and $\sigma_{3}<1$, the system (2) is locally asymptotically stable at the steady-state $O_{1}\text{ }(\bar{x}_{1},\bar{y}_{1},\bar{z}_{1})$.

**(2)** If $\sigma_{3}>\max\{1,\text{ }1+\frac{\sigma_{1}\text{ }-1}{K}\}$, the system (2) is locally asymptotically stable at the steady-state $O_{2}\text{ }(\bar{x}_{2},\bar{y}_{2},\bar{z}_{2})$.

**(3)** If $\sigma_{1}>1$,$\sigma_{3}<\text{ }1+\frac{\sigma_{1}\text{ }-1}{K}$ and $\alpha_{2}<C$, the system (2) is locally asymptotically stable at the steady-state $O_{3}\text{ }(\bar{x}_{3},\bar{y}_{3},\bar{z}_{3})$, where

$C=\frac{\sigma_{1}}{\sigma_{1}-1}\cdot[1+\alpha_{4}-\frac{K^{2}\sigma_{3}\alpha_{4}}{(K+\sigma_{1}-1)^{2}}]\cdot\frac{(K+\sigma_{1}-1)-\frac{K^{2}\sigma_{3}}{K+\sigma_{1}-1}}{K+\sigma_{1}-1-K\sigma_{3}}$.

Proof: (1) The Jacobi matrix of model (2) at $O_{1}\text{ }(\bar{x}_{1},\bar{y}_{1},\bar{z}_{1})$ is

$J_{O_{1}}=\left(\begin{matrix} (\sigma_{1}-1)\alpha_{2} & 0 & 0 \\ 1 & (\sigma_{3}-1)\alpha_{4} & 0 \\ 0 & 1 & -1 \end{matrix}\right)$.

Its corresponding characteristic equation is

$$\begin{matrix} \left|\lambda I-J_{O_{1}}\right|=\left|\begin{matrix} \lambda-(\sigma_{1}-1)\alpha_{2} & 0 & 0 \\ -1 & \lambda-(\sigma_{3}-1)\alpha_{4} & 0 \\ 0 & -1 & \lambda+1 \end{matrix}\right| \\ \text{ }=[\lambda-(\sigma_{1}-1)\alpha_{2}][\lambda-(\sigma_{3}-1)\alpha_{4}](\lambda+1)=0 \end{matrix}$$

We can see from the above equation that the roots of the equation have negative real parts if $\sigma_{1}<1$ and $\sigma_{3}<1$, which show that the model (2) is locally asymptotically stable at the steady-state $O_{1}\text{ }(\bar{x}_{1},\bar{y}_{1},\bar{z}_{1})$ **( See Figure S1(A) for the simulation)**.

(2) The Jacobi matrix of model (2) at $O_{2}\text{ }(\bar{x}_{2},\bar{y}_{2},\bar{z}_{2})$ is

$$J_{O_{2}}=\left(\begin{matrix} \frac{\sigma_{1}\alpha_{2}}{1+\bar{z}_{2}}-\alpha_{2} & 0 & 0 \\ 1 & \frac{K^{2}\sigma_{3}\alpha_{4}}{(K+\bar{y}_{2})^{2}}-\alpha_{4} & 0 \\ 0 & 1 & -1 \end{matrix}\right)$$

Its corresponding characteristic equation is

$$\left|\lambda I-J_{O_{2}}\right|=\left|\begin{matrix} \lambda+\alpha_{2}-\frac{\sigma_{1}\alpha_{2}}{1+\bar{z}_{2}} & 0 & 0 \\ -1 & \lambda+\alpha_{4}-\frac{K^{2}\sigma_{3}\alpha_{4}}{(K+\bar{y}_{2})^{2}} & 0 \\ 0 & -1 & \lambda+1 \end{matrix}\right|$$

$$\begin{matrix} \text{ }=(\lambda+\alpha_{2}-\frac{\sigma_{1}\alpha_{2}}{1+\bar{z}_{2}})(\lambda+\alpha_{4}-\frac{K^{2}\sigma_{3}\alpha_{4}}{(K+\bar{y}_{2})^{2}})(\lambda+1) \\ \text{ }=(\lambda+\alpha_{2}-\frac{\sigma_{1}\alpha_{2}}{K\sigma_{3}+1-K})(\lambda+\alpha_{4}-\frac{\alpha_{4}}{\sigma_{3}})(\lambda+1)=0 \end{matrix}$$

We can see from the above equation that the roots of the equation have negative real parts if $\sigma_{3}>\text{ }1+\frac{\sigma_{1}-1}{K}$ and $\sigma_{3}>1$, which show that the model (2) is locally asymptotically stable at non-negative steady-state $O_{2}\text{ }(\bar{x}_{2},\bar{y}_{2},\bar{z}_{2})$**( See Figure S1(B) for the simulation)**.

(3) The Jacobi matrix of system (4) at $O_{3}\text{ }(\bar{x}_{3},\bar{y}_{3},\bar{z}_{3})$ is

$$J_{O_{3}}=\left(\begin{matrix} \frac{\sigma_{1}\alpha_{2}}{1+\bar{z}_{3}}-\alpha_{2} & 0 & -\frac{\sigma_{1}\alpha_{2}\bar{x}_{3}}{(1+\bar{z}_{3})^{2}} \\ 1 & \frac{K^{2}\sigma_{3}\alpha_{4}}{(K+\bar{y}_{3})^{2}}-\alpha_{4} & 0 \\ 0 & 1 & -1 \end{matrix}\right)$$

Its corresponding characteristic equation is

$$\left|\lambda I-J_{O_{3}}\right|=\left|\begin{matrix} \lambda+\alpha_{2}-\frac{\sigma_{1}\alpha_{2}}{1+\bar{z}_{3}} & 0 & \frac{\sigma_{1}\alpha_{2}\bar{x}_{3}}{(1+\bar{z}_{3})^{2}} \\ -1 & \lambda+\alpha_{4}-\frac{K^{2}\sigma_{3}\alpha_{4}}{(K+\bar{y}_{3})^{2}} & 0 \\ 0 & -1 & \lambda+1 \end{matrix}\right|$$

$$\begin{matrix} \text{ }=(\lambda+\alpha_{2}-\frac{\sigma_{1}\alpha_{2}}{1+\bar{z}_{3}})[\lambda+\alpha_{4}-\frac{K^{2}\sigma_{3}\alpha_{4}}{(K+\bar{y}_{3})^{2}}](\lambda+1)+\frac{\sigma_{1}\alpha_{2}\bar{x}_{3}}{(1+\bar{z}_{3})^{2}} \\ \text{ }=\lambda(\lambda+1)[\lambda+\alpha_{4}-\frac{K^{2}\sigma_{3}\alpha_{4}}{(K+\text{ }\sigma_{1}-1)^{2}}]+\frac{\alpha_{2}\alpha_{4}(\sigma_{1}-1)(K+\sigma_{1}-1-K\sigma_{3})}{\sigma_{1}(K+\sigma_{1}-1)}=0 \end{matrix}$$

i.e.

$\begin{matrix} \lambda^{3}+[1+\alpha_{4}-\frac{K^{2}\sigma_{3}\alpha_{4}}{(K+\sigma_{1}-1)^{2}}]\lambda^{2}+[\alpha_{4}-\frac{K^{2}\sigma_{3}\alpha_{4}}{(K+\sigma_{1}-1)^{2}}]\lambda\\ \text{ }+\frac{\alpha_{2}\alpha_{4}(\sigma_{1}-1)(K+\sigma_{1}-1-K\sigma_{3})}{\sigma_{1}(K+\sigma_{1}-1)}=0 \end{matrix}$ (A1)

When $\sigma_{1}>1$,$\sigma_{3}<1+\frac{\sigma_{1}-1}{K}$ and $\alpha_{2}<C$, we can induce that $(K+\sigma_{1}-1)^{2}>K^{2}\sigma_{3}$ and

$$[1+\alpha_{4}-\frac{K^{2}\sigma_{3}\alpha_{4}}{(K+\sigma_{1}-1)^{2}}][1-\frac{K^{2}\sigma_{3}}{(K+\sigma_{1}-1)^{2}}]>\frac{\alpha_{2}(\sigma_{1}-1)(K+\sigma_{1}-1-K\sigma_{3})}{\sigma_{1}(K+\sigma_{1}-1)}$$

Then all conditions in Routh-Hurwitz criterion are satisfied, accordingly, all roots of the equation have negative real parts **( See Figure S1(C) for the simulation)**. ▐

We can infer the following sufficient conditions because it is easily found that *C* > 1.

**Corollary S1**：If $\sigma_{1}>1$, $\sigma_{3}<1$ and $\alpha_{2}\leq1$, the system (2) is locally asymptotically stable at the non-negative steady-state $O_{3}\text{ }(\bar{x}_{3},\bar{y}_{3},\bar{z}_{3})$. ▐

As it can be seen from the above analysis, when $\sigma_{1}$, $\sigma_{3}$ and $K$ are fixed, the change of $\alpha_{2}$ should have impact on the stability of the non-negative steady-state $O_{3}(\bar{x}_{3},\bar{y}_{3},\bar{z}_{3})$. Taking into account that our model has a negative feedback, we conjecture that the increase of $\alpha_{2}$ will destabilize the equilibrium point $O_{3}(\bar{x}_{3},\bar{y}_{3},\bar{z}_{3})$ if $\alpha_{2}$ is large enough, and usually we find that periodic solution (self-oscillation) can arise from equilibrium by Hopf bifurcation (Similar to *n*_1_=*n*_2_=2).

**Theorem S2.** If RVD($\alpha_{2}$) is viewed as parameter, then a Hopf bifurcation occurs as $\alpha_{2}$ passes through $C$ for the model (2).

Proof: To facilitate the discussion below, equation (A1) is denoted by

$\lambda^{3}+p\lambda^{2}+q\lambda+r=0$ (A2)

Where$p=1+\alpha_{4}-\frac{K^{2}\sigma_{3}\alpha_{4}}{(K+\sigma_{1}-1)^{2}}$, $q=\alpha_{4}-\frac{K^{2}\sigma_{3}\alpha_{4}}{(K+\sigma_{1}-1)^{2}}$, $r=\frac{\alpha_{2}\alpha_{4}(\sigma_{1}-1)(K+\sigma_{1}-1-K\sigma_{3})}{\sigma_{1}(K+\sigma_{1}-1)}$

According to the Hopf bifurcation theorem and the Routh-Hurwitz criteria, a Hopf bifurcation occurs at the following conditions $p>0$，$q>0$，$r>0$ and$pq-r=0$. Let

$H(\lambda(\alpha_{2}),\alpha_{2})=\lambda^{3}+p\lambda^{2}+q\lambda+r$ (A3)

The eigenvalues of Eq. (A2) at $\alpha_{2}=C$ are

$\lambda_{1}=-p<0$, $\lambda_{2,3}=\pm i\sqrt{q}$

To illustrate the Hopf bifurcation of the system at $\alpha_{2}=C$, we need to show

$\mathrm{Re}(\frac{d\lambda_{2,3}(C)}{d\alpha_{2}})>0$.

Differentiating (A3) yields $\frac{d\lambda}{d\alpha_{2}}=-\frac{\partial H/\partial\alpha_{2}}{\partial H/\partial\lambda}=-\frac{p'\lambda^{2}+q'\lambda+r'}{3\lambda^{2}+2p\lambda+q}$.

Where $p'=\frac{dp}{d\alpha_{2}}=0$，$q'=\frac{dq}{d\alpha_{2}}=0$，$r'=\frac{dr}{d\alpha_{2}}=\frac{\alpha_{4}(\sigma_{1}-1)(K+\sigma_{1}-1-K\sigma_{3})}{\sigma_{1}(K+\sigma_{1}-1)}>0$.

The complex eigenvalues of Eq. (A3) $\lambda_{2}$ and $\lambda_{3}$ occur in pairs, so $\lambda_{2}$ is only used to calculate the following

$$\frac{d\lambda_{2}}{d\alpha_{2}}=-\frac{\partial H/\partial\alpha_{2}}{\partial H/\partial\lambda_{2}} =\frac{2qr'+2p\sqrt{q}r'i}{4q^{2}+4p^{2}q}$$

We find the real part of the above equation

$$\mathrm{Re}(\frac{d\lambda_{2}(C)}{d\alpha_{2}})=\frac{2qr'}{4q^{2}+4p^{2}q}=\frac{2q}{4q^{2}+4p^{2}q}\frac{(\sigma_{1}-1)[\alpha_{4}(K+\sigma_{1}-1)-K\sigma_{3}\alpha_{4}]}{\sigma_{1}(K+\sigma_{1}-1)}>0$$

Therefore, we show that a Hopf bifurcation occurs as $\alpha_{2}$ passes through $C$ for the system (2) **(See Figure S1(D)&(E) , Figure S2(F)&(G) for the simulation and Figure S3 for the bifurcation graph)**. ▐

**Theorem S3** When $\tau_{i}=0,i=1,2,3,4,5$ and *n*_1_=*n*_2_=2, the model (2) exists three non-negative stable steady states under certain conditions

$O_{1}^{'}\text{ }(\bar{x}_{1}^{'},\bar{y}_{1}^{'},\bar{z}_{1}^{'})$: $\bar{x}_{1}^{'}=\bar{y}_{1}^{'}=\bar{z}_{1}^{'}=0$

$O_{2}^{'}\text{ }(\bar{x}_{2}^{'},\bar{y}_{2}^{'},\bar{z}_{2}^{'})$: $\bar{x}_{2}^{'}=0,\text{ }\bar{z}_{2}^{'}=\bar{y}_{2}^{'}=\frac{K(\sigma_{3}+\sqrt{\sigma_{3}^{2}-4})}{2}$

$O_{3}^{'}\text{ }(\bar{x}_{3}^{'},\bar{y}_{3}^{'},\bar{z}_{3}^{'})$: $\bar{x}_{3}^{'}=\sqrt{\sigma_{1}-1}(1-\frac{K\sigma_{3}\sqrt{\sigma_{1}-1}}{K^{2}+\sigma_{1}-1})\alpha_{4},\text{ }\bar{z}_{3}^{'}=\bar{y}_{3}^{'}=\sqrt{\sigma_{1}-1}$

and an unstable non-negative steady-state

$O_{2}^{' '}\text{ }(\bar{x}_{2}^{' '},\bar{y}_{2}^{' '},\bar{z}_{2}^{' '})$: $\bar{x}_{2}^{' '}=0,\text{ }\bar{z}_{2}^{' '}=\bar{y}_{2}^{' '}=\frac{K(\sigma_{3}-\sqrt{\sigma_{3}^{2}-4})}{2}$

(1) If $\sigma_{1}<1$, the system (2) is locally asymptotically stable at the steady-state $O_{1}\text{' }(\bar{x}_{1}',\bar{y}_{1}',\bar{z}_{1}')$.

(2) If $\sigma_{3}>2$ and $\sigma_{3}>\frac{K^{2}+\text{ }\sigma_{1}-1}{K\sqrt{\sigma_{1}-1}}\text{ }(\sigma_{1}\geq1+K^{2})$, the system (2) is locally asymptotically stable at the non-negative steady-state $O_{2}\text{' }(\bar{x}_{2}',\bar{y}_{2}',\bar{z}_{2}')$.

(3) the non-negative steady-state $O_{2}^{' '}\text{ }(\bar{x}_{2}^{' '},\bar{y}_{2}^{' '},\bar{z}_{2}^{' '})$ is unstable.

(4) If $\sigma_{1}>1$，$\sigma_{3}<\min\{\frac{K^{2}+\text{ }\sigma_{1}-1}{K\sqrt{\sigma_{1}-1}},\text{ }\frac{(K^{2}+\text{ }\sigma_{1}-1)^{2}}{2K^{3}\sqrt{\sigma_{1}-1}}\}$ and $\alpha_{2}<C'$, the system (2) is locally asymptotically stable at the non-negative steady-state $O_{3}\text{' }(\bar{x}_{3}',\bar{y}_{3}',\bar{z}_{3}')$, where $C'=\frac{\sigma_{1}}{2(\sigma_{1}-1)}\cdot[1+\alpha_{4}-\frac{2K^{3}\sigma_{3}\alpha_{4}\sqrt{\sigma_{1}-1}}{(K^{2}+\text{ }\sigma_{1}-1)^{2}}]\cdot\frac{(K^{2}+\text{ }\sigma_{1}-1)-\frac{2K^{3}\sigma_{3}\sqrt{\sigma_{1}-1}}{K^{2}+\text{ }\sigma_{1}-1}}{K^{2}+\sigma_{1}-1-K\sigma_{3}\sqrt{\sigma_{1}-1}}$.

Proof. (1) The Jacobi matrix at the steady-state $O_{1}\text{' }(\bar{x}_{1}',\bar{y}_{1}',\bar{z}_{1}')$ is

$$J_{O_{1}'}=\left(\begin{matrix} (\sigma_{1}-1)\alpha_{2} & 0 & 0 \\ 1 & -\alpha_{4} & 0 \\ 0 & 1 & -1 \end{matrix}\right)$$

The corresponding characteristic equation is

$\left|\lambda I-J_{O_{1}'}\right|=\left|\begin{matrix} \lambda-(\sigma_{1}-1)\alpha_{2} & 0 & 0 \\ -1 & \lambda+\alpha_{4} & 0 \\ 0 & -1 & \lambda+1 \end{matrix}\right|=[\lambda-(\sigma_{1}-1)\alpha_{2}](\lambda+\alpha_{4})(\lambda+1)=0$We can see from the above equation that the roots of the equation have negative real parts if $\sigma_{1}<1$, which show that the system (2) is locally asymptotically stable at the steady-state $O_{1}\text{' }(\bar{x}_{1}',\bar{y}_{1}',\bar{z}_{1}')$ **(See Figure S2 (B) and (D) for the simulation)**.

(2) The Jacobi matrix at the non-negative steady-state $O_{2}\text{' }(\bar{x}_{2}',\bar{y}_{2}',\bar{z}_{2}')$ is

$$J_{O_{2}'}=\left(\begin{matrix} \frac{\sigma_{1}\alpha_{2}}{1+\bar{z}'_{2}^{2}}-\alpha_{2} & 0 & 0 \\ 1 & \frac{2K^{3}\sigma_{3}\alpha_{4}\bar{y}'_{2}}{(K^{2}+\bar{y}'_{2}^{2})^{2}}-\alpha_{4} & 0 \\ 0 & 1 & -1 \end{matrix}\right)$$

The corresponding characteristic equation is

$$\left|\lambda I-J_{O_{2}'}\right|=\left|\begin{matrix} \lambda+\alpha_{2}-\frac{\sigma_{1}\alpha_{2}}{1+\bar{z}'_{2}^{2}} & 0 & 0 \\ -1 & \lambda+\alpha_{4}-\frac{2K^{3}\sigma_{3}\alpha_{4}\bar{y}'_{2}}{(K^{2}+\bar{y}'_{2}^{2})^{2}} & 0 \\ 0 & -1 & \lambda+1 \end{matrix}\right|$$

$$\begin{matrix} \text{ }=(\lambda+\alpha_{2}-\frac{\sigma_{1}\alpha_{2}}{1+\bar{z}'_{2}^{2}})[\lambda+\alpha_{4}-\frac{2K^{3}\sigma_{3}\alpha_{4}\bar{y}'_{2}}{(K^{2}+\bar{y}'_{2}^{2})^{2}}](\lambda+1) \\ \text{ }=(\lambda+\alpha_{2}-\frac{\sigma_{1}\alpha_{2}}{1+\bar{y}'_{2}^{2}})(\lambda+\alpha_{4}-\frac{2K^{2}\alpha_{4}}{K^{2}+\bar{y}'_{2}^{2}})(\lambda+1)=0 \end{matrix}$$

We can see from the above equation that the roots of the equation have negative real parts If $\sigma_{3}>2$ and $\sigma_{1}<1+\frac{K^{2}[(\sigma_{3}^{2}-2)+\sigma_{3}\sqrt{\sigma_{3}^{2}-4}]}{2}$， i.e. $\sigma_{3}>\frac{K^{2}+\text{ }\sigma_{1}-1}{K\sqrt{\sigma_{1}-1}}\text{ }(\sigma_{1}\geq1+K^{2})$, which show that the system (2) is locally asymptotically stable at the non-negative steady-state (Figure S2C; Figure S4A and C).

(3) Similarly, the discussion for $O_{2}^{' '}\text{ }(\bar{x}_{2}^{' '},\bar{y}_{2}^{' '},\bar{z}_{2}^{' '})$ shows that the conditions of $\sigma_{3}<2$ required for system stability will conflict with the conditions of $\sigma_{3}>2$ for non-negative of $O_{2}^{' '}\text{ }(\bar{x}_{2}^{' '},\bar{y}_{2}^{' '},\bar{z}_{2}^{' '})$, indicating that under its non-negative meaning, the corresponding Jacobi matrix has positive eigenvalues. Therefore does, the non-negative equilibrium $O_{2}^{' '}\text{ }(\bar{x}_{2}^{' '},\bar{y}_{2}^{' '},\bar{z}_{2}^{' '})$ is unstable.

(4) The Jacobi matrix at the non-negative steady-state $O_{3}\text{' }(\bar{x}_{3}',\bar{y}_{3}',\bar{z}_{3}')$ is

$$J_{O_{3}'}=\left(\begin{matrix} \frac{\sigma_{1}\alpha_{2}}{1+\bar{z}'_{3}^{2}}-\alpha_{2} & 0 & -\frac{2\sigma_{1}\alpha_{2}\bar{x}'_{3}\bar{z}'_{3}}{(1+\bar{z}'_{3}^{2})^{2}} \\ 1 & \frac{2K^{3}\sigma_{3}\alpha_{4}\bar{y}'_{3}}{(K^{2}+\bar{y}'_{3}^{2})^{2}}-\alpha_{4} & 0 \\ 0 & 1 & -1 \end{matrix}\right)$$

The corresponding characteristic equation is

$\left|\lambda I-J_{O_{3}'}\right|=\left|\begin{matrix} \lambda+\alpha_{2}-\frac{\sigma_{1}\alpha_{2}}{1+\bar{z}'_{3}^{2}} & 0 & \frac{2\sigma_{1}\alpha_{2}\bar{x}'_{3}\bar{z}'_{3}}{(1+\bar{z}'_{3}^{2})^{2}} \\ -1 & \lambda+\alpha_{4}-\frac{2K^{3}\sigma_{3}\alpha_{4}\bar{y}'_{3}}{(K^{2}+\bar{y}'_{3}^{2})^{2}} & 0 \\ 0 & -1 & \lambda+1 \end{matrix}\right|$ $\begin{matrix} \text{ }=(\lambda+\alpha_{2}-\frac{\sigma_{1}\alpha_{2}}{1+\bar{z}'_{3}^{2}})[\lambda+\alpha_{4}-\frac{2K^{3}\sigma_{3}\alpha_{4}\bar{y}'_{3}}{(K^{2}+\bar{y}'_{3}^{2})^{2}}](\lambda+1)+\frac{2\sigma_{1}\alpha_{2}\bar{x}'_{3}\bar{z}'_{3}}{(1+\bar{z}'_{3}^{2})^{2}} \\ \text{ }=\lambda(\lambda+1)[\lambda+\alpha_{4}-\frac{2K^{3}\sigma_{3}\alpha_{4}\sqrt{\sigma_{1}-1}}{(K^{2}+\text{ }\sigma_{1}-1)^{2}}]+\frac{2\alpha_{2}\alpha_{4}(\sigma_{1}-1)(K^{2}+\sigma_{1}-1-K\sigma_{3}\sqrt{\sigma_{1}-1})}{\sigma_{1}(K^{2}+\text{ }\sigma_{1}-1)}=0 \end{matrix}$i.e.

$$\begin{matrix} \lambda^{3}+[1+\alpha_{4}-\frac{2K^{3}\sigma_{3}\alpha_{4}\sqrt{\sigma_{1}-1}}{(K^{2}+\text{ }\sigma_{1}-1)^{2}}]\lambda^{2}+[\alpha_{4}-\frac{2K^{3}\sigma_{3}\alpha_{4}\sqrt{\sigma_{1}-1}}{(K^{2}+\text{ }\sigma_{1}-1)^{2}}]\lambda\\ \text{ }+\frac{2\alpha_{2}\alpha_{4}(\sigma_{1}-1)(K^{2}+\sigma_{1}-1-K\sigma_{3}\sqrt{\sigma_{1}-1})}{\sigma_{1}(K^{2}+\text{ }\sigma_{1}-1)}=0 \end{matrix}$$

Due to the conditions of $\bar{x}_{3}'>0,\text{ }\bar{y}_{3}'>0,\text{ }\bar{z}_{3}'>0$, we know from the Routh-Hurwitz criterion that the roots of the equation have negative real parts, which show that the system (2) is locally asymptotically stable at the non-negative steady-state $O_{3}\text{' }(\bar{x}_{3}',\bar{y}_{3}',\bar{z}_{3}')$, if $(K^{2}+\text{ }\sigma_{1}-1)^{2}>2K^{3}\sigma_{3}\sqrt{\sigma_{1}-1}$ and

$[1+\alpha_{4}-\frac{2K^{3}\sigma_{3}\alpha_{4}\sqrt{\sigma_{1}-1}}{(K^{2}+\text{ }\sigma_{1}-1)^{2}}][1-\frac{2K^{3}\sigma_{3}\sqrt{\sigma_{1}-1}}{(K^{2}+\text{ }\sigma_{1}-1)^{2}}]>\frac{2\alpha_{2}(\sigma_{1}-1)(K^{2}+\sigma_{1}-1-K\sigma_{3}\sqrt{\sigma_{1}-1})}{\sigma_{1}(K^{2}+\text{ }\sigma_{1}-1)}$ .

We can obtain $\sigma_{3}<\frac{K^{2}+\text{ }\sigma_{1}-1}{K\sqrt{\sigma_{1}-1}}$ and $\sigma_{3}<\frac{(K^{2}+\text{ }\sigma_{1}-1)^{2}}{2K^{3}\sqrt{\sigma_{1}-1}}$ from $\bar{x}'_{3}>0$ and $(K^{2}+\text{ }\sigma_{1}-1)^{2}>2K^{3}\sigma_{3}\sqrt{\sigma_{1}-1}$, respectively.

Note that if $\text{ }\bar{x}'_{3}>0,\text{ }\bar{y}'_{3}>0$，and then$\sigma_{1}>1\text{ }$，$K^{2}+\text{ }\sigma_{1}-1>K\sigma_{3}\sqrt{\sigma_{1}-1}$. Therefore, if $\alpha_{2}<C'$, where

$C'=\frac{\sigma_{1}}{2(\sigma_{1}-1)}\cdot[1+\alpha_{4}-\frac{2K^{3}\sigma_{3}\alpha_{4}\sqrt{\sigma_{1}-1}}{(K^{2}+\text{ }\sigma_{1}-1)^{2}}]\cdot\frac{(K^{2}+\text{ }\sigma_{1}-1)-\frac{2K^{3}\sigma_{3}\sqrt{\sigma_{1}-1}}{K^{2}+\text{ }\sigma_{1}-1}}{K^{2}+\sigma_{1}-1-K\sigma_{3}\sqrt{\sigma_{1}-1}}$,

we have

$[1+\alpha_{4}-\frac{2K^{3}\sigma_{3}\alpha_{4}\sqrt{\sigma_{1}-1}}{(K^{2}+\text{ }\sigma_{1}-1)^{2}}][1-\frac{2K^{3}\sigma_{3}\sqrt{\sigma_{1}-1}}{(K^{2}+\text{ }\sigma_{1}-1)^{2}}]>\frac{2\alpha_{2}(\sigma_{1}-1)(K^{2}+\sigma_{1}-1-K\sigma_{3}\sqrt{\sigma_{1}-1})}{\sigma_{1}(K^{2}+\text{ }\sigma_{1}-1)}$.

Then all conditions in Routh-Hurwitz criterion are satisfied, accordingly, all roots of the equation have negative real parts, which show that the system (2) is locally asymptotically stable at non-negative steady-state $O_{3}\text{' }(\bar{x}_{3}',\bar{y}_{3}',\bar{z}_{3}')$ **(Figure S2(E) and Figure S4(B) for the simulation).** ▐

Assume that *n*_1_=*n*_2_=1, and then we can obtain the following system (A4) from (2):

$$\left\{\begin{matrix} \frac{dx}{dt}=\frac{\sigma_{1}\alpha_{2}x(t-\tau_{1})}{1+z(t-\tau_{5})}-\alpha_{2}x(t) \\ \frac{dy}{dt}=x(t-\tau_{2})+\frac{\sigma_{2}\alpha_{4}y(t-\tau_{4})}{K+y(t-\tau_{4})}-\alpha_{4}y(t) \\ \frac{dz}{dt}=y(t-\tau_{3})-z(t) \end{matrix}\right.\text{ (A4)}$$

**Theorem S4: If the system (A4) without time delays is local stable at the steady state *O*_1_(0, 0, 0), then the system (A4) with time delays still maintains the local stability at the steady state *O*_1_(0, 0, 0), no matter how the time delays are changed.**

Proof: We linearize system (A4) around $O_{1}\text{ }(0,0,0)$, and obtain the following system:

$$\left\{\begin{matrix} \frac{dx}{dt}=\sigma_{1}\alpha_{2}x(t-\tau_{1})-\alpha_{2}x(t) \\ \frac{dy}{dt}=x(t-\tau_{2})+\frac{\sigma_{2}\alpha_{4}}{K}y(t-\tau_{4})-\alpha_{4}y(t) \\ \frac{dz}{dt}=y(t-\tau_{3})-z(t) \end{matrix}\right.$$

The corresponding characteristic equation is

$$\left|\lambda I-J_{O_{1}}^{\dagger}\right|=\left|\begin{matrix} \lambda+\alpha_{2}-\sigma_{1}\alpha_{2}e^{-\tau_{1}\lambda} & 0 & 0 \\ -e^{-\tau_{2}\lambda} & \lambda+\alpha_{4}-\frac{\sigma_{2}\alpha_{4}}{K}e^{-\tau_{4}\lambda} & 0 \\ 0 & -e^{-\tau_{3}\lambda} & \lambda+1 \end{matrix}\right|$$

$$\text{ }=(\lambda+\alpha_{2}-\sigma_{1}\alpha_{2}e^{-\tau_{1}\lambda})(\lambda+\alpha_{4}-\frac{\sigma_{2}\alpha_{4}}{K}e^{-\tau_{4}\lambda})(\lambda+1)=0\text{ }(\text{A5})$$

Obviously, $\tau_{2}$, $\tau_{3}$and $\tau_{5}$ don’t affect the stability because they don’t appear in the characteristic equation (A5). So we need only investigate that whether $\tau_{1}$ or $\tau_{4}$ changes the stability of the system (A4) at the steady state $O_{1}\text{ }(0,0,0)$.

From **Theorem** S1, when $\tau_{1}=\tau_{4}=0$ and $\sigma_{1}<1$, $\sigma_{2}<K$, the roots of the above characteristic equation have negative real parts, which show that the system (A4) is locally asymptotically stable at equilibrium point $O_{1}\text{ }(0,0,0)$.

Obviously, there is a real root of $\lambda_{1}=-1$ for characteristic equation (A5). So if the equation (A5) has complex roots, they must come in pairs. Assume that $\lambda=\alpha\pm i\beta$. We Substitute $\lambda+\alpha_{2}-\sigma_{1}\alpha_{2}e^{-\tau_{1}\lambda}=0$ (or $\lambda+\alpha_{4}-\frac{\sigma_{2}\alpha_{4}}{K}e^{-\tau_{4}\lambda}=0$) with $\lambda=\alpha\pm i\beta$, then

$\alpha+i\beta+\alpha_{2}-\sigma_{1}\alpha_{2}e^{-\tau_{1}(\alpha+i\beta)}=0$，

i.e. $(\alpha+\alpha_{2}-\sigma_{1}\alpha_{2}e^{-\tau_{1}\alpha}\cos\tau_{1}\beta)+i(\beta+\sigma_{1}\alpha_{2}e^{-\tau_{1}\alpha}\sin\tau_{1}\beta)=0$

Its real part is $\alpha+\alpha_{2}-\sigma_{1}\alpha_{2}e^{-\tau_{1}\alpha}\cos\tau_{1}\beta=0$

then $\cos\tau_{1}\beta=\frac{\alpha+\alpha_{2}}{\sigma_{1}\alpha_{2}e^{-\tau_{1}\alpha}}$

If the system (A4) without time delays is local stable at $O_{1}\text{ }(0,0,0)$, there is the condition of $\sigma_{1}<1$. So, if $\alpha\geq0$, then $\alpha+\alpha_{2}>\sigma_{1}\alpha_{2}\geq\sigma_{1}\alpha_{2}e^{-\tau_{1}\alpha}$, i.e. $\frac{\alpha+\alpha_{2}}{\sigma_{1}\alpha_{2}e^{-\tau_{1}\alpha}}>1$，but $\left|\cos\tau_{1}\beta\right|\leq1$. It is contradictory! This shows that $\alpha<0$. Therefore, all characteristic roots of system (A4) have negative real parts, then it is locally stable at the steady state $O_{1}\text{ }(0,0,0)$. ▐

**Theorem S5: If the system (A4) without time delays is local stable at the steady state** $O_{2}(0,\sigma_{2}-K,\sigma_{2}-K)$ **then the system (A4) with time delays still maintains the local stability at the steady state** $O_{2}(0,\sigma_{2}-K,\sigma_{2}-K)$**, no matter how the time delays are changed.**

Proof: Consider system (A4) and the steady-state $O_{2}(0,\sigma_{2}-K,\sigma_{2}-K)$. Let us introduce new variables $\tilde{x}(t)=x(t)$, $\tilde{y}(t)=y(t)-(\sigma_{2}-K)$ and $\tilde{z}(t)=z(t)-(\sigma_{2}-K)$. After rewriting system (A4), We linearize it around $O_{2}(0,\sigma_{2}-K,\sigma_{2}-K)$, and obtain the following system:

$$\left\{\begin{matrix} \frac{d\tilde{x}}{dt}=\frac{\sigma_{1}\alpha_{2}}{1+\sigma_{2}-K}\tilde{x}(t-\tau_{1})-\alpha_{2}\tilde{x}(t) \\ \frac{d\tilde{y}}{dt}=\tilde{x}(t-\tau_{2})+\frac{K\alpha_{4}}{\sigma_{2}}\tilde{y}(t-\tau_{4})-\alpha_{4}\tilde{y}(t) \\ \frac{d\tilde{z}}{dt}=\tilde{y}(t-\tau_{3})-\tilde{z}(t) \end{matrix}\right.$$

The corresponding characteristic equation is

$$\left|\lambda I-J_{O_{2}}^{\dagger}\right|=\left|\begin{matrix} \lambda+\alpha_{2}-\frac{\sigma_{1}\alpha_{2}e^{-\tau_{1}\lambda}}{1+\sigma_{2}-K} & 0 & 0 \\ -e^{-\tau_{2}\lambda} & \lambda+\alpha_{4}-\frac{K\alpha_{4}e^{-\tau_{4}\lambda}}{\sigma_{2}} & 0 \\ 0 & -e^{-\tau_{3}\lambda} & \lambda+1 \end{matrix}\right|$$

$$\text{ }=(\lambda+\alpha_{2}-\frac{\sigma_{1}\alpha_{2}}{1+\sigma_{2}-K}e^{-\tau_{1}\lambda})(\lambda+\alpha_{4}-\frac{K\alpha_{4}}{\sigma_{2}}e^{-\tau_{4}\lambda})(\lambda+1)=0$$

Similar to the discussions in **Theorem S4**, all characteristic roots of system (A4) have negative real parts, then , it is locally stable at the steady state.▐

It is much more sophisticated for the impact of delays on the third steady state $O_{3}\text{ }((\sigma_{1}-1)(1-\frac{\sigma_{2}}{K+\sigma_{1}-1})\alpha_{4},\sigma_{1}-1,\sigma_{1}-1)$ than the one on the first steady state $O_{1}\text{ }(0,0,0)$ or the second steady state $O_{2}(0,\sigma_{2}-K,\sigma_{2}-K)$. To obtain the results of delays on the third steady state $O_{3}$, we introduce a lemma which can ensure that the cubic equation must have a single positive real root if its relations of coefficients would be satisfied [[[1]](#endnote-2)]:

**Lemma 1:** For $x^{3}+c_{1}x^{2}+c_{2}x+c_{3}=0$, if $c_{1}>0$ and $c_{3}>0$, then a necessary and sufficient conditions to ensure at least a single positive root are (1) $c_{2}<0$; (2) $\Delta<0$. Where $\Delta=4c_{2}^{3}-c_{1}^{2}c_{2}^{2}+4c_{1}^{3}c_{3}-18c_{1}c_{2}c_{3}+27c_{3}^{2}$.

**Remark:** For a cubic function $f(x)=x^{3}+c_{1}x^{2}+c_{2}x+c_{3}$, if it has a single positive root, indicating that the function has a only single positive root $x_{+}$ or two unequal positive root $x_{+\_small}$ and $x_{+}$ ($x_{+\_small}$<$x_{+}$), then the cubic function at $x_{+}$ has a positive derivative, i.e. $f'(x_{+})>0$. And we note that $\Delta<0$ must be satisfied if $c_{2}$ is small enough.

To facilitate the discussion about the impact of the delays on the third steady state $O_{3}$, we introduce new variables $\tilde{x}(t)=x(t)-(\sigma_{1}-1)(1-\frac{\sigma_{2}}{K+\sigma_{1}-1})\alpha_{4}$, $\tilde{y}(t)=y(t)-(\sigma_{1}-1)$ and $\tilde{z}(t)=z(t)-(\sigma_{1}-1)$. After rewriting system (A4), We linearize it around $O_{3}$, and obtain the following system:

$$\left\{\begin{matrix} \frac{d\tilde{x}}{dt}=\alpha_{2}\tilde{x}(t-\tau_{1})-\frac{\alpha_{2}\bar{x}_{3}}{\sigma_{1}}\tilde{z}(t-\tau_{5})-\alpha_{2}\tilde{x}(t) \\ \frac{d\tilde{y}}{dt}=\tilde{x}(t-\tau_{2})+\frac{K\sigma_{2}\alpha_{4}}{(K+\sigma_{1}-1)^{2}}\tilde{y}(t-\tau_{4})-\alpha_{4}\tilde{y}(t) \\ \frac{d\tilde{z}}{dt}=\tilde{y}(t-\tau_{3})-\tilde{z}(t) \end{matrix}\right.$$

The corresponding characteristic equation is

$\begin{matrix} \left|\lambda I-J_{O_{3}}^{\dagger}\right|=\left|\begin{matrix} \lambda+\alpha_{2}-\alpha_{2}e^{-\tau_{1}\lambda} & 0 & \frac{\alpha_{2}\bar{x}_{3}}{\sigma_{1}}e^{-\tau_{5}\lambda} \\ -e^{-\tau_{2}\lambda} & \lambda+\alpha_{4}-\frac{K\sigma_{2}\alpha_{4}}{(K+\sigma_{1}-1)^{2}}e^{-\tau_{4}\lambda} & 0 \\ 0 & -e^{-\tau_{3}\lambda} & \lambda+1 \end{matrix}\right| \\ \text{ }=(\lambda+\alpha_{2}-\alpha_{2}e^{-\tau_{1}\lambda})(\lambda+\alpha_{4}-\frac{K\sigma_{2}\alpha_{4}}{(K+\sigma_{1}-1)^{2}}e^{-\tau_{4}\lambda})(\lambda+1)+\frac{\alpha_{2}\bar{x}_{3}}{\sigma_{1}}e^{-(\tau_{2}+\tau_{3}+\tau_{5})\lambda} \\ \text{ }=(\lambda+\alpha_{2}-\alpha_{2}e^{-\tau_{1}\lambda})(\lambda+\alpha_{4}-\gamma e^{-\tau_{4}\lambda})(\lambda+1)+ke^{-(\tau_{2}+\tau_{3}+\tau_{5})\lambda} \\ \text{ }=0\text{ }(\text{A}6) \end{matrix}$where

$$\gamma=\frac{K\sigma_{2}\alpha_{4}}{(K+\sigma_{1}-1)^{2}}\text{, }k=\frac{\alpha_{2}\bar{x}_{3}}{\sigma_{1}}=\frac{\alpha_{2}\alpha_{4}(\sigma_{1}-1)(K+\sigma_{1}-1-\sigma_{2})}{\sigma_{1}(K+\sigma_{1}-1)}$$

It is well known that the stability of steady states depends on the sign of the real parts of the roots of Eq. (A6) [[[2]](#endnote-3), [[3]](#endnote-4)]. From Eq. (A6), we have

$$\begin{matrix} \lambda^{3}+(1+\alpha_{2}+\alpha_{4})\lambda^{2}+(\alpha_{2}+\alpha_{4}+\alpha_{2}\alpha_{4})\lambda+\alpha_{2}\alpha_{4} \\ \text{ }=\alpha_{2}e^{-\tau_{1}\lambda}[\lambda^{2}+(1+\alpha_{4})\lambda+\alpha_{4}]\text{+}\gamma e^{-\tau_{4}\lambda}[\lambda^{2}+(1+\alpha_{2})\lambda+\alpha_{2}] \\ \text{ }-\alpha_{2}\gamma e^{-(\tau_{1}+\tau_{4})\lambda}(\lambda+1)-e^{-(\tau_{2}+\tau_{3}+\tau_{5})\lambda}k\text{ }(\text{A}7) \end{matrix}$$

Let $\lambda=i\beta\text{ }(\text{ }\beta\in R\text{ })$，and rewrite Eq. (A7) in terms of its real and imaginary parts as

$$\begin{matrix} \alpha_{2}\alpha_{4}-(1+\alpha_{2}+\alpha_{4})\beta^{2}=-(\alpha_{2}\beta^{2}-\alpha_{2}\alpha_{4})\cos\tau_{1}\beta+(\alpha_{2}+\alpha_{2}\alpha_{4})\beta\sin\tau_{1}\beta\\ \text{ }-(\gamma\beta^{2}-\gamma\alpha_{2})\cos\tau_{4}\beta+\gamma(1+\alpha_{2})\beta\sin\tau_{4}\beta\\ \text{ }-\gamma\alpha_{2}\cos(\tau_{1}+\tau_{4})\beta-\gamma\alpha_{2}\beta\sin(\tau_{1}+\tau_{4})\beta\\ \text{ }-k\cos(\tau_{2}+\tau_{3}+\tau_{5})\beta\text{ }(\text{A}8) \end{matrix}$$

$$\begin{matrix} (\alpha_{2}+\alpha_{4}+\alpha_{2}\alpha_{4})\beta-\beta^{3}=(\alpha_{2}\beta^{2}-\alpha_{2}\alpha_{4})\sin\tau_{1}\beta+(\alpha_{2}+\alpha_{2}\alpha_{4})\beta\cos\tau_{1}\beta\\ \text{ }+(\gamma\beta^{2}-\gamma\alpha_{2})\sin\tau_{4}\beta+\gamma(1+\alpha_{2})\beta\cos\tau_{4}\beta\\ \text{ }+\gamma\alpha_{2}\sin(\tau_{1}+\tau_{4})\beta-\gamma\alpha_{2}\beta\cos(\tau_{1}+\tau_{4})\beta\\ \text{ }+k\sin(\tau_{2}+\tau_{3}+\tau_{5})\beta\text{ }(\text{A}9) \end{matrix}$$

**Theorem S6: For the stable steady state $O_{3}$, all delays (*τ*_1_, *τ*_2_, *τ*_3_, *τ*_4_ and *τ*_5_) can induce a Hopf bifurcation under certain conditions:**

**(1) When *τ*_1_ (or *τ*_4_) is viewed as parameter and the other delays are 0, a Hopf bifurcation occurs as $\tau_{1}$ passes through some $\tau_{1B}$ if $\alpha_{2}>C_{tau1}$ and $\Delta_{tau1}<0$ (or as $\tau_{4}$ passes through some $\tau_{4B}$ if $\alpha_{2}>C_{tau4}$ and $\Delta_{tau4}<0$).**

**(2) When *τ*_2_ (or *τ*_3_, or *τ*_5_) is viewed as parameter and the other delays are 0, a Hopf bifurcation occurs as $\tau_{2}$ (or $\tau_{3}$, or $\tau_{5}$) passes through some $\tau_{2B}$(or $\tau_{3B}$, or $\tau_{5B}$).**

**Where**

$C_{tau1}=\sqrt{\frac{\alpha_{4}-\gamma}{2(1+\alpha_{4}-\gamma)}\cdot C+\frac{1}{4}(\alpha_{4}-\gamma+1)^{2}}-\frac{1}{2}(\alpha_{4}-\gamma+1)$

$C_{tau4}=\frac{\alpha_{4}+\gamma}{2(1+\alpha_{4}-\gamma)(1+\alpha_{4})}\cdot C$

$\begin{matrix} \Delta_{tau1}=4[(\alpha_{4}-\gamma)^{2}-2k(\alpha_{2}+\alpha_{4}-\gamma+1)]^{3}-[(\alpha_{4}-\gamma)^{2}+1]^{2}[(\alpha_{4}-\gamma)^{2}-2k(\alpha_{2}+\alpha_{4}-\gamma+1)]^{2} \\ \text{ }+4[(\alpha_{4}-\gamma)^{2}+1]^{3}(2r_{1}+k)k-18[(\alpha_{4}-\gamma)^{2}+1][(\alpha_{4}-\gamma)^{2}-2k(\alpha_{2}+\alpha_{4}-\gamma+1)](2r_{1}+k)k \\ \text{ }+27(2r_{1}+k)^{2}k^{2} \end{matrix}$ $\begin{matrix} \Delta_{tau4}=4[\alpha_{4}^{2}-\gamma^{2}-2k(1+\alpha_{4})]^{3}-(1+\alpha_{4}^{2}-\gamma^{2})^{2}[\alpha_{4}^{2}-\gamma^{2}-2k(1+\alpha_{4})]^{2}+4(1+\alpha_{4}^{2}-\gamma^{2})^{3}k^{2} \\ \text{ }-18(1+\alpha_{4}^{2}-\gamma^{2})[\alpha_{4}^{2}-\gamma^{2}-2k(1+\alpha_{4})]k^{2}+27k^{4} \end{matrix}$

Proof: (1) Firstly, we set $\tau_{2}=\tau_{3}=\tau_{4}=\tau_{5}=0$，then (A8) and (A9) are reduced to

$$r_{0}-p_{0}\beta^{2}=-(p_{1}\beta^{2}-r_{1})\cos\tau_{1}\beta+q_{1}\beta\sin\tau_{1}\beta\text{ }(\text{A}10)$$

$$-\beta^{3}+q_{0}\beta=(p_{1}\beta^{2}-r_{1})\sin\tau_{1}\beta+q_{1}\beta\cos\tau_{1}\beta\text{ }(\text{A}11)$$

where

$$\begin{matrix} p_{0}=\alpha_{2}+\alpha_{4}-\gamma+1,\text{ }q_{0}=\alpha_{2}(1+\alpha_{4}-\gamma)+\alpha_{4}-\gamma,\text{ }r_{0}=\alpha_{2}(\alpha_{4}-\gamma)+k \\ p_{1}=\alpha_{2},\text{ }q_{1}=\alpha_{2}(1+\alpha_{4}-\gamma),\text{ }r_{1}=\alpha_{2}(\alpha_{4}-\gamma) \end{matrix}$$

If the first bifurcation point is $(\beta_{1B}^{0},\tau_{1B}^{0})$, then the other bifurcation points $(\beta_{1B},\tau_{1B})$ satisfy

$\beta_{1B}\tau_{1B}=\beta_{1B}^{0}\tau_{1B}^{0}+2n\pi$，（$n=1,2,3\cdot\cdot\cdot,\infty$）

By squaring the two equations (A10) and (A11) respectively and then adding them, it follows that

$\beta^{6}+(p_{0}^{2}-2q_{0}-p_{1}^{2})\beta^{4}+(q_{0}^{2}+2p_{1}r_{1}-2p_{.0}r_{0}-q_{1}^{2})\beta^{2}+(2r_{1}+k)k=0$ (A12)

Here we note that this is a cubic equation about $\beta^{2}$ and whether the cubic equation has positive real root is related with the coefficients.

From (A12), we have

$f(\beta^{2})=\beta^{6}+[(\alpha_{4}-\gamma)^{2}+1]\beta^{4}+[(\alpha_{4}-\gamma)^{2}-2k(\alpha_{2}+\alpha_{4}-\gamma+1)]\beta^{2}+(2r_{1}+k)k$ (A13)

Let $c_{1\beta}=(\alpha_{4}-\gamma)^{2}+1$, $c_{2\beta}=(\alpha_{4}-\gamma)^{2}-2k(\alpha_{2}+\alpha_{4}-\gamma+1)$ and $c_{3\beta}=(2r_{1}+k)k$, it is obvious that$c_{1\beta}>0$ and $c_{3\beta}>0$.

From lemma 1, we know that a necessary and sufficient conditions to ensure at least a single positive root for the function (A13) are (1) $c_{2\beta}<0$; (2) $\Delta_{tau1}<0$. And we prove that if $\alpha_{2}>C_{tau1}$, then $c_{2\beta}<0$, i.e. $(\alpha_{4}-\gamma)^{2}-2k(\alpha_{2}+\alpha_{4}-\gamma+1)<0$

i.e. $[\alpha_{4}-\frac{K\sigma_{2}\alpha_{4}}{(K+\sigma_{1}-1)^{2}}]^{2}-2(\alpha_{2}+\alpha_{4}-\gamma+1)\frac{\alpha_{2}\alpha_{4}(\sigma_{1}-1)(K+\sigma_{1}-1-\sigma_{2})}{\sigma_{1}(K+\sigma_{1}-1)}<0$

i.e. $2\alpha_{2}(\alpha_{2}+\alpha_{4}-\gamma+1)>\frac{\alpha_{4}-\gamma}{1+\alpha_{4}-\gamma}\cdot\frac{\sigma_{1}}{\sigma_{1}-1}\cdot[1+\alpha_{4}-\frac{K\sigma_{2}\alpha_{4}}{(K+\sigma_{1}-1)^{2}}]\cdot\frac{(K+\sigma_{1}-1)-\frac{K\sigma_{2}}{(K+\sigma_{1}-1)}}{K+\sigma_{1}-1-\sigma_{2}}$i.e. $\alpha_{2}(\alpha_{2}+\alpha_{4}-\gamma+1)>\frac{\alpha_{4}-\gamma}{2(1+\alpha_{4}-\gamma)}\cdot C$

i.e. $\alpha_{2}^{2}+(\alpha_{4}-\gamma+1)\alpha_{2}-\frac{\alpha_{4}-\gamma}{2(1+\alpha_{4}-\gamma)}\cdot C>0$

So, when $\alpha_{2}>\sqrt{\frac{\alpha_{4}-\gamma}{2(1+\alpha_{4}-\gamma)}\cdot C+\frac{1}{4}(\alpha_{4}-\gamma+1)^{2}}-\frac{1}{2}(\alpha_{4}-\gamma+1)$, i.e. $\alpha_{2}>C_{tau1}$, then the above inequality is established.

Then, there is a single positive root of Eq. (A12), supposing $\beta_{1B}$ is the single positive root of Eq. (A12), at which the cubic function about $\beta^{2}$ has a positive derivative.

From Eq. (A7), then

$$\lambda^{3}+p_{0}\lambda^{2}+q_{0}\lambda+r_{0}=e^{-\tau_{1}\lambda}(p_{1}\lambda^{2}+q_{1}\lambda+r_{1})$$

Denote

$$H(\lambda,\tau_{1})=\lambda^{3}+p_{0}\lambda^{2}+q_{0}\lambda+r_{0}-e^{-\tau_{1}\lambda}(p_{1}\lambda^{2}+q_{1}\lambda+r_{1})$$

Let $\lambda\left(\tau_{1}\right)=\alpha\left(\tau_{1}\right)+i\beta\left(\tau_{1}\right)$ be root of Eq. (A12) with $\alpha\left(\tau_{1b}\right)=0$ and $\beta\left(\tau_{1b}\right)=\beta_{1b}$, then

$$\frac{d\lambda}{d\tau_{1}}=-\frac{\partial H/\partial\tau_{1}}{\partial H/\partial\lambda}=\frac{-\lambda e^{-\tau_{1}\lambda}(p_{1}\lambda^{2}+q_{1}\lambda+r_{1})}{3\lambda^{2}+2p_{0}\lambda+q_{0}+\tau_{1}e^{-\tau_{1}\lambda}(p_{1}\lambda^{2}+q_{1}\lambda+r_{1})-e^{-\tau_{1}\lambda}(2p_{1}\lambda+q_{1})}$$

Evaluating the real part of this equation at $\tau_{1}=\tau_{1B}$ and setting $\lambda=i\beta_{1B}$ yield

$$\begin{matrix} \left.\frac{d\alpha}{d\tau_{1}}\right|_{\tau_{1}=\tau_{1b}}=\mathrm{Re}(\left.\frac{d\lambda}{d\tau_{1}})\right|_{\tau_{1}=\tau_{1B}} \\ \text{ } =\frac{\beta_{1B}^{2}[3\beta_{1B}^{4}+2(p_{0}^{2}-2q_{0}-p_{1}^{2})\beta_{1B}^{2}+q_{0}^{2}+2p_{1}r_{1}-2p_{0}r_{0}-q_{1}^{2}]}{M_{1}^{2}+N_{1}^{2}} \end{matrix}$$

Where

$$M_{1}=-3\beta_{1B}^{2}+q_{0}+[\tau_{1B}r_{1}-q_{1}-\tau_{1B}p_{1}\beta_{1B}^{2}]\cos\beta_{1B}\tau_{1B}+(\tau_{1B}q_{1}-2p_{1})\beta_{1B}\sin\beta_{1B}\tau_{1B}$$

$$N_{1}=2p_{0}\beta_{1B}+(\tau_{1B}q_{1}-2p_{1})\beta_{1B}\cos\beta_{1B}\tau_{1B}+[\tau_{1B}p_{1}\beta_{1B}^{2}-\tau_{1B}r_{1}+q_{1}]\sin\beta_{1B}\tau_{1B}$$

Let $\eta=\beta^{2}$，then Eq. (A12) can be reduced to

$$g(\eta)=\eta^{3}+(p_{0}^{2}-2q_{0}-p_{1}^{2})\eta^{2}+(q_{0}^{2}+2p_{1}r_{1}-2p_{.0}r_{0}-q_{1}^{2})\eta+(2r_{1}+k)k=0$$

Then

$$g'(\eta)=3\eta^{2}+2(p_{0}^{2}-2q_{0}-p_{1}^{2})\eta+(q_{0}^{2}+2p_{1}r_{1}-2p_{.0}r_{0}-q_{1}^{2})$$

If $\beta_{1B}$ is the least positive simple root of Eq. (A12), then

$$\frac{dg}{d\eta}\left|_{\eta=\beta_{1B}^{2}}\right.>0$$

Hence,

$$\frac{d\alpha}{d\tau_{1}}\left|_{\tau_{1}=\tau_{1B}}\right.=\mathrm{Re}\left(\frac{d\lambda}{d\tau_{1}}\right)\left|_{\tau_{1}=\tau_{1B}}=\frac{\beta_{1B}^{2}g'\left(\beta_{1B}^{2}\right)}{M_{1}^{2}+N_{1}^{2}}>0\right.$$

According to the Hopf bifurcation theorem, a Hopf bifurcation occurs as $\tau_{1}$ passes through $\tau_{1B}$.

Secondly, we set $\tau_{1}=\tau_{2}=\tau_{3}=\tau_{5}=0$. Similar to $\tau_{1}$, we have the result that when *τ*_4_ is viewed as parameter and the other delays are 0, a Hopf bifurcation occurs as $\tau_{4}$ passes through some $\tau_{4B}$ if $\alpha_{2}>C_{tau4}$ and $\Delta_{tau4}<0$.

(2) For $\tau_{2}$, we set$\tau_{1}=\tau_{3}=\tau_{4}=\tau_{5}=0$，then (A8) and (A9) are reduced to

$(p_{1}-p_{0})\beta^{2}=-k\cos\tau_{2}\beta$ (A14)

$-\beta^{3}+(q_{0}-q_{1})\beta=k\sin\tau_{2}\beta$ (A15)

If the first bifurcation point is $(\beta_{2B}^{0},\tau_{2B}^{0})$, then the other bifurcation points $(\beta_{2B},\tau_{2B})$ satisfy

$\beta_{2B}\tau_{2B}=\beta_{2B}^{0}\tau_{2B}^{0}+2n\pi$，（$n=1,2,3\cdot\cdot\cdot,\infty$）

By squaring the two equations (A14) and (A15) respectively and then adding them, it follows that

$\beta^{6}+[(p_{1}-p_{0})^{2}-2(q_{0}-q_{1})]\beta^{4}+(q_{0}-q_{1})^{2}\beta^{2}-k^{2}=0$ (A16)

Here we note that this is a cubic equation about $\beta^{2}$ and that the left side is positive for large of $\beta^{2}$ and negative for $\beta=0$($-k^{2}<0$), i.e. Eq. (A16) has at least one positive real root. Therefore, similar to the proof of (1), we have the following conclusion:

When *τ*_2_ (or *τ*_3_, or *τ*_5_) is viewed as parameter and the other delays are 0, a Hopf bifurcation occurs as $\tau_{2}$ (or $\tau_{3}$, or $\tau_{5}$)passes through some $\tau_{2B}$(or $\tau_{3B}$, or $\tau_{5B}$). ▐

**Remark:** (1) We note that $\alpha_{2}>C_{tau1}$ can be satisfied under $\alpha_{2}<C$ because $C>\sqrt{\frac{\alpha_{4}-\gamma}{2(1+\alpha_{4}-\gamma)}\cdot C+\frac{1}{4}(\alpha_{4}-\gamma+1)^{2}}-\frac{1}{2}(\alpha_{4}-\gamma+1)$ ($∵\text{ }C>1\text{ }and\text{ }C>\alpha_{4}-\gamma+1$). This indicates that the delay time $\tau_{1}$ may cause Hopf bifurcation or remain stable for the stable steady state $O_{3}$ according to Theorem S5 and Theorem S1(3).

(2) Similarly, the condition of $\alpha_{2}>C_{tau4}$ can be satisfied under $\alpha_{2}<C$ because $C>\frac{\alpha_{4}+\gamma}{2(1+\alpha_{4}-\gamma)(1+\alpha_{4})}\cdot C$ ($∵\text{ }\alpha_{4}>\gamma$). This indicates that the delay time $\tau_{4}$ may cause Hopf bifurcation or remain stable for the stable steady state $O_{3}$ according to Theorem S5 and Theorem S1(3).

For small delay $(\tau<<1)$, linear stability analysis is very convenient to find the bifurcation point. Thus, let $e^{-\lambda\tau}\approx1-\lambda\tau$，then , the characteristic equation (A6) becomes

$\lambda^{3}+p\lambda^{2}+q\lambda+r=0$ (A17)

where

$$p=\frac{(1+\alpha_{2}\tau_{1})(1+\gamma\tau_{4})+(1+\alpha_{2}\tau_{1})(\alpha_{4}-\gamma)+k(\tau_{2}\tau_{3}+\tau_{2}\tau_{5}+\tau_{3}\tau_{5})}{(1+\alpha_{2}\tau_{1})(1+\gamma\tau_{4})-k\tau_{2}\tau_{3}\tau_{5}}$$

$$q=\frac{(1+\alpha_{2}\tau_{1})(\alpha_{4}-\gamma)-k(\tau_{2}+\tau_{3}+\tau_{5})}{(1+\alpha_{2}\tau_{1})(1+\gamma\tau_{4})-k\tau_{2}\tau_{3}\tau_{5}}$$

$$r=\frac{k}{(1+\alpha_{2}\tau_{1})(1+\gamma\tau_{4})-k\tau_{2}\tau_{3}\tau_{5}}$$

By the Hopf bifurcation theorem and the Routh-Hurwitz criteria, a Hopf bifurcation occurs at $\tau_{i}=\tau_{ib},\text{ (}i=1,2,3,4,5)$ where

$p>0$，$q>0$，$r>0$，$pq-r=0$

Let

$H(\lambda(\tau_{i}),\tau_{i})=\lambda^{3}+p\lambda^{2}+q\lambda+r$ (A18)

The eigenvalues of Eq. (A17) at $\tau_{i}=\tau_{ib}$ are

$\lambda_{1}=-p<0$, $\lambda_{2,3}=\pm i\sqrt{q}$

To illustrate the Hopf bifurcation of the system at $\tau_{i}=\tau_{ib}$, we should show $\mathrm{Re}(\frac{d\lambda_{2}(\tau_{ib})}{d\tau_{i}})>0$. Differentiating (A18) yields

$$\frac{d\lambda}{d\tau_{i}}=-\frac{\partial H/\partial\tau_{i}}{\partial H/\partial\lambda}=-\frac{p'\lambda^{2}+q'\lambda+r'}{3\lambda^{2}+2p\lambda+q}$$

where $p'=\frac{dp}{d\tau_{i}}$，$q'=\frac{dq}{d\tau_{i}}$，$r'=\frac{dr}{d\tau_{i}}$，$i=1,\text{ }2,\text{ }3,\text{ 4},\text{ 5}$.

The complex eigenvalues of Eq. (A17) $\lambda_{2}$ and $\lambda_{3}$ occur in pairs, so $\lambda_{2}$ need only be used to calculate the following

$$\frac{d\lambda_{2}(\tau_{ib})}{d\tau_{i}}=-\frac{\partial H/\partial\tau_{i}}{\partial H/\partial\lambda_{2}} =\frac{2q(r'-p'q)-2q'pq+i[2q'q\sqrt{q}+2p\sqrt{q}(r'-p'q)]}{4q^{2}+4p^{2}q}$$

The real part of the above

$$\mathrm{Re}(\frac{d\lambda_{2}(\tau_{ib})}{d\tau_{i}})=\frac{2q(r'-p'q)-2q'pq}{4q^{2}+4p^{2}q}=\frac{r'-p'q-pq'}{2q+2p^{2}}$$

The real part is always positive if the following conditions are valid:

$r'>p'q+pq'$ (A19)

So, the inequality (A19) is sufficient to have a positive slope of the real part of the eigenvalue $\lambda_{2}(\tau_{ib})$. This fact (according to the Hopf bifurcation theorem) might guarantee the bifurcation to a limit cycle for $\tau_{i}=\tau_{ib}$ $i=1,\text{ }2,\text{ }3,\text{ 4},\text{ 5}$.

References

1. [?] Khan Q. Hopf bifurcation in multiparty political systems with time delay in switching. Appl.Math.Lett., 2000, 13:45-52. [↑](#endnote-ref-2)
2. [?] Nikolov S and Petrov V. Time delay model of RNA Silencing. Journal of Mechanics in Medicine and Biology, 2007, 7: 297-314. [↑](#endnote-ref-3)
3. [?] Galach M. Dynamics of the tumor-immune system competition-the effect of time delay. Int. J. Appl. Math. Comput. Sci., 2003, 13: 395–406. [↑](#endnote-ref-4)
